# A targeted sequencing panel identifies rare damaging variants in multiple genes in the cranial neural tube defect, anencephaly

**DOI:** 10.1111/cge.13189

**Published:** 2018-02-11

**Authors:** M. Ishida, T. Cullup, C. Boustred, C. James, J. Docker, C. English, GOSgene, N. Lench, A.J. Copp, G.E. Moore, N.D.E. Greene, P. Stanier

**Affiliations:** ^1^ Genetics and Genomic Medicine, UCL Great Ormond Street Institute of Child Health University College London London UK; ^2^ Great Ormond Street Hospital North East Thames Regional Genetics Service Laboratories London UK; ^3^ Institute of Genetic Medicine Newcastle University Newcastle upon Tyne UK; ^4^ Congenica Ltd Cambridge UK; ^5^ Developmental Biology and Cancer, UCL Great Ormond Street Institute of Child Health University College London London UK

**Keywords:** anencephaly, craniorachischisis, molecular diagnosis, neural tube defects, targeted exome sequencing

## Abstract

Neural tube defects (NTDs) affecting the brain (anencephaly) are lethal before or at birth, whereas lower spinal defects (spina bifida) may lead to lifelong neurological handicap. Collectively, NTDs rank among the most common birth defects worldwide. This study focuses on anencephaly, which despite having a similar frequency to spina bifida and being the most common type of NTD observed in mouse models, has had more limited inclusion in genetic studies. A genetic influence is strongly implicated in determining risk of NTDs and a molecular diagnosis is of fundamental importance to families both in terms of understanding the origin of the condition and for managing future pregnancies. Here we used a custom panel of 191 NTD candidate genes to screen 90 patients with cranial NTDs (*n* = 85 anencephaly and *n* = 5 craniorachischisis) with a targeted exome sequencing platform. After filtering and comparing to our in‐house control exome database (*N* = 509), we identified 397 rare variants (minor allele frequency, MAF < 1%), 21 of which were previously unreported and predicted damaging. This included 1 frameshift (*PDGFRA*), 2 stop‐gained (*MAT1A*; *NOS2*) and 18 missense variations. Together with evidence for oligogenic inheritance, this study provides new information on the possible genetic causation of anencephaly.

## 1 INTRODUCTION

Neural tube defects (NTDs) are a group of anomalies in which closure of the future brain and/or spinal cord of the developing embryo is abnormal, resulting in a birth defect. Collectively, they rank among the most common human birth defects worldwide with a prevalence of 0.5 to 2 per 1000 pregnancies. NTDs can be broadly divided into primary and secondary neurulation defects.^1^ The former group are evenly split between anencephaly, in which the cranial neural tube fails to close exposing the mid and hind brain region and spina bifida aperta where the low spinal neural tube remains open. Much more rarely the neural folds remain open along the entire body axis, which is called craniorachischisis. Anencephaly and craniorachischisis result in stillbirth or infant mortality, while spinal defects can be associated with lifelong neurological disability. It is important to note that approximately 85% of open NTD cases in the United Kingdom are terminated.^2^ Given the high prevalence and traumatic consequences for affected individuals and their families, the identification of the precise genetic, biochemical and cellular factors involved is a high priority.

NTDs are generally considered multifactorial with both genetic and environmental factors implicated in their etiology. Although most NTDs occur sporadically, there is a strong genetic component with a heritability estimate of up to 70%.^3^ Despite this, the molecular basis in humans has proven difficult to resolve. This may be attributed to the high degree of heterogeneity between unrelated sporadic cases, incomplete penetrance and the general paucity of large individual families displaying Mendelian inheritance. The complex molecular requirements for normal neural tube closure are well illustrated by the occurrence of NTDs in more than 200 different mouse genetic models.^4^

It has been estimated that up to 70% of potential NTD cases are preventable by the mother taking periconceptional supplements of folic acid.^5^, ^6^ However, evidence for causal mutations in genes regulating folate metabolism remain tenuous.^7^ In humans, planar cell polarity (PCP) gene variants have been reported in heterozygous form throughout the NTD phenotypic spectrum. When tested, these variants are mostly found in an unaffected parent, prompting speculation regarding incomplete penetrance effects, which may be explained by co‐inheritance of additional variants in other closely related or interacting genes that remain undetected using the methodologies employed.^8^ This genetic interaction is evident in NTD mouse models doubly heterozygous for PCP genes,^9^ suggesting that a comprehensive study of PCP genes in humans would be a valuable exercise.

The patient cohorts in most candidate gene studies contain mixed primary and secondary neurulation phenotypes, typically including only a small number of craniorachischisis or anencephalic cases. There is therefore a relative lack of information concerning the genetic basis of anencephaly in humans. In contrast, exencephaly, the developmental forerunner of anencephaly, is present in more than 90% of mouse mutants that transmit NTDs in a Mendelian fashion. It is also striking that the primary folate‐sensitive NTD in mouse models is exencephaly, with less supporting evidence for primary prevention of mouse spina bifida by folic acid supplementation.^4^, ^10^, ^11^ Even so, it is clear that both anencephaly and spina bifida can be prevented by folic acid in humans.^5^, ^12^ In addition, there is a marked excess in females in anencephaly and craniorachischisis compared to spina bifida.^1^ Collectively, these data might suggest that genetic factors implicated in anencephaly and craniorachischisis could be different from those implicated in spina bifida cases.

In this study, we have carried out a comprehensive, targeted exome sequencing of fetuses with anencephaly using a panel of 191 genes with biological relevance to NTDs. Unlike the traditional gene‐by‐gene search method, this allowed us to test the hypothesis that multiple hits in different, but closely related genes may combine to provide an oligogenic explanation for NTD risk.

## 2 MATERIALS AND METHODS

### 2.1 NTD and healthy control human DNA samples

The sample group for this study comprised 85 fetuses with anencephaly and 4 with craniorachischisis that underwent pregnancy termination following prenatal diagnosis by ultrasound in the north east of England between 1992 and 2011. Although folic acid supplements were recommended from 1991 onwards there was no mandatory folate fortification of food for this population and folic acid supplement use, typically 400 μg/day, has been estimated to have an uptake of up to around 30%.^13^ Fibroblast samples were collected by the Northern Genetics Service following signed consent and approved for research under The Newcastle Upon Tyne Hospitals NHS Trust Ethical Committee. DNA was extracted from fetal fibroblasts using the DNeasy Blood and Tissue kit (Qiagen, Hilden, Germany) following the manufacturer's protocol. One further craniorachischisis sample obtained from Hammersmith Hospital, London, UK was also included, making a total of 90 NTD samples. No parental samples were available for the NTD cases. Control DNA samples were extracted from whole blood obtained from 6 healthy white Europeans (2 trios) in the Moore cohort.^14^ The samples were collected under the guidelines of the Hammersmith and Queen Charlotte's and Chelsea Hospitals’ NHS Trusts Research Ethics Committee (registration no. 2001/6029).

### 2.2 Selection of NTD candidate genes

The targeted gene panel for NTDs included 191 genes that were selected by one of several criteria. These included genes that encode enzymes of folate one‐carbon metabolism or components of the PCP or related cell polarity pathways that are implicated in normal neurulation in animal models. Additional members of these pathways were added even where a role in neurulation has not yet been directly indicated and also multiple gene family members, for example, *CELSR1*, *CELSR2* and *CELSR3*, irrespective of whether there was a prior established link with NTDs. The panel design also included a number of prominent genes underlying NTDs in well‐studied mouse models (eg, *PAX3* and *ZIC3*; see gene panel list in Table S1 in Appendix S3, Supporting information).

### 2.3 Target enrichment library preparation and sequencing

Custom capture libraries were generated from 3 μg gDNA using the SureSelectXT Reagent kit (Agilent Technologies, Santa Clara, California). RNA baits (Table S1 in Appendix S3) were designed using SureDesign (Agilent Technologies), with an estimated average coverage of 98.2% of targeted regions. Samples were sequenced with MiSeq Reagent kit v3 (150 cycle; 2 × 75 bp) on the MiSeq Sequencing System (Illumina, San Diego, California).

### 2.4 Sequence alignment, variant annotation and filtering

An in‐house pipeline was used for the sequence alignment, variant calling and annotation. FASTQ files were trimmed with Cutadapt^15^ and the sequencing reads were aligned to the human reference genome (hg19) using the Burrows‐Wheeler Aligner (v0.6.1‐r104)^16^. SAMtools (v0.1.18)^17^ was used to generate mpileup files for variant calling with VarScan2 (v2.3.6)^18^ to generate VCF files. Only variants with >30 read depths, >0.2 variant frequency, and average quality score of >20 were called. Candidate variants were manually checked on the integrative genomics viewer. Principal component analysis was used to assess sample relatedness (Methods in Appendix S2).

### 2.5 Variant filtering and prioritization

Variants were annotated with Ensembl Variant Effect Predictor (v73: http://www.ensembl.org/info/docs/tools/vep/index.html). Provean,^19^ SIFT,^20^ PolyPhen2,^21^ Condel,^22^ REVEL^23^ and MutationTaster^24^ were used for predicting the effects of variants. REVEL score >0.5 were used as threshold for classifying pathogenic variants. Intronic, synonymous, 3′ and 5′ UTR, up‐ and downstream variants were removed. Rare variants were defined as minor allele frequency (MAF) <1% in the Exome Aggregation Consortium (ExAC) Database.^25^ Variants that were specific to NTD patients and not present in the capture control (*N* = 6), in‐house exome controls (*N* = 509) and in the dbSNP,^26^ 1000 Genomes Project,^27^ ExAC, and the Genome Aggregation Database (gnomAD)^25^ were labeled “Novel,” and were verified by Sanger sequencing (Methods in Appendix S2).

### 2.6 Mutation burden analysis

The burden of carrying multiple rare/novel variants within the 191 candidate genes was compared between the NTD and the ExAC cohorts. To allow an unbiased gene‐level comparison, the sequencing coverage of the targeted regions was analyzed between the 2 cohorts. The exon coordinates for the canonical transcripts of all 191 genes were extracted as a Browser Extensible Data (BED) file from the University of California Santa Cruz (UCSC) database, which was used as the reference sequence for target regions. The percentage coverage of the targeted bases at ≥×30 read depths was calculated using Picard (http://broadinstitute.github.io/picard/) for the NTD samples, and the coverage information for the ExAC data was downloaded from the ExAC database. The gene‐level coverage between the cohorts was compared using Student's *t* test (2‐tailed). Because the sample‐level information is not available at the ExAC database, the number of each variant was compared in the burden analysis for consistency as described in D'Alessandro et al.^28^ Only rare/novel variants predicted to be damaging were used for further analysis. Fisher's exact test (2‐tailed) was used to assess the enrichment of rare/novel variants on the genes showing comparable sequence coverage. Bonferroni correction was performed by multiplying the each *P*‐value with the number of genes used in the test (*n* = 13).

## 3 RESULTS

**Figure 1:**
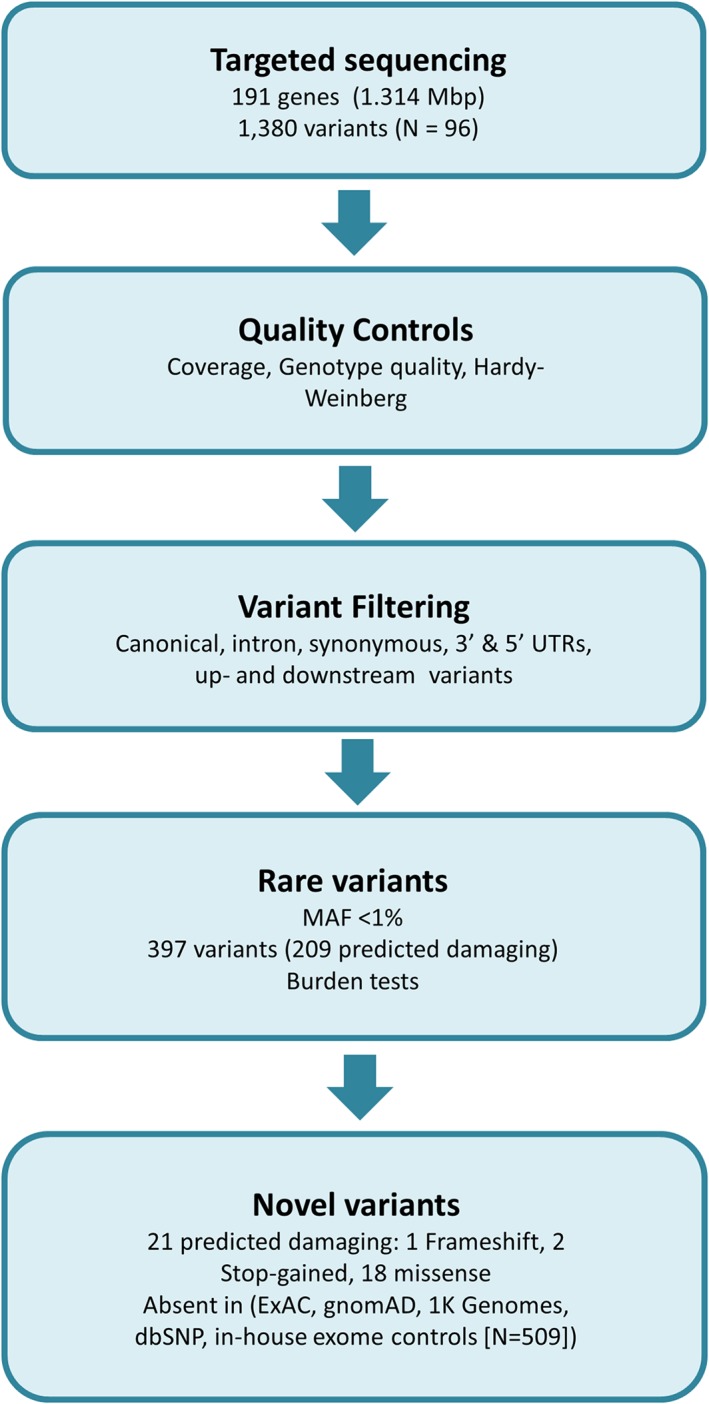
Flow chart showing the capture variant analysis pathway. Following sequencing, data is processed through a pipeline which provides quality control followed by a series of filters to identify plausible causative variants. MAF, minor allele frequency; UTR, untranslated region

On average, 97.5% of bases were covered at ≥×30 read depth across 191 genes, indicating a high targeting efficiency. A mean target coverage depth of ×110 was achieved with more than 96% of reads having Phred quality score greater than Q30 (corresponds to 99.9% base call accuracy). A total of 1380 variants, which passed quality control, were identified of which 1359 were single nucleotide variants (SNVs) and 21 were insertions/deletions (Indels) (Figure 1).

### 3.1 Relatedness analysis

Principal component analysis identified 3 samples (52F03, 00F133 and 389F07) with higher variation than the rest of the samples which might be suggestive of different ethnic background (Figure S1 in Appendix S1). Apart from the healthy control trios (child‐mother‐father) included in the capture sequencing, kinship analysis did not indicate relatedness between any of the samples (Figure S2 in Appendix S1), including 2 NTD cases which carried an identical extremely rare variant in the *CELSR1* gene (described below).

### 3.2 Previously reported pathogenic mutations associated with NTDs

One craniorachischisis/exomphalos sample (01F29A) was found to carry a pathogenic missense variant (c.676G>A; p.Ala226Thr; rs104894043) in the Sonic Hedgehog (*SHH*) gene, which was previously identified in an autosomal‐dominant holoprosencephaly 3 (HPE3) patient (OMIM: 142945).^29^ HPE3 is characterized by midline brain and craniofacial abnormalities, and it is possible that this variant either contributes to the NTD, whilst other characteristics of HPE are masked by the severity of the midline craniorachischisis/exomphalos phenotype in this individual. One fetus with anencephaly (735F97) carried a rare missense mutation (c.2852C>A; p.Ser951Tyr; rs147472391) in glycine decarboxylase (*GLDC*), which was previously reported as one of the causative mutated alleles in a compound heterozygous patient with the autosomal recessive disorder, non‐ketotic hyperglycinemia (NKH, OMIM: 605899).^30^ *GLDC* encodes a component of the glycine cleavage system (GCS) in mitochondrial folate one‐carbon metabolism, which has previously been implicated in both mouse and human NTDs.^10^, ^11^, ^31^, ^32^ Another individual with anencephaly (706F07) was heterozygous for a missense variant (c.200C>T; p.Thr67Ile; rs28941785) in the cystathionine gamma‐lyase (*CTH*) gene, which causes cystathioninuria in a recessive form.^33^

### 3.3 Unreported and rare variants

After filtering out intron, 3′ and 5′ UTR and synonymous variants, a total of 397 rare (MAF < 1%, including novel) variants were identified in 89/90 NTD cases from 110/191 of the panel of NTD candidate genes. Of these, 209 variants were predicted to be damaging by at least one of the mutation effect predictors described in Methods (summarized in Table S2 in Appendix S3). Following an additional filter to include our in‐house exomes (*n* = 509) and publically available data (1000Genome, dbSNP, ExAC and gnomAD), there remained 21 novel variants that were predicted to be damaging (1 frameshift, 2 stop‐gained and 18 missense; Table 1).

**Table 1:** List of novel variants predicted damaging

ID	Position	R	A	Gene	cDNA	Protein	Type	Provean	SIFT	PolyPhen	Condel	REVEL	Mutation Taster
283F06	chr1:11856442	G	A	*MTHFR*	c.601C>T	p.His201Tyr	Missense	Deleterious	Deleterious	Probably damaging	Deleterious	0.932	Disease causing
436F98	chr1:236208932	T	C	*NID1*	c.577A>G	p.Ile193Val	Missense	Neutral	Tolerated	Benign	Neutral	0.217	Disease causing
00F576	chr3:125828823	T	G	*ALDH1L1*	c.2341A>C	p.Thr781Pro	Missense	Deleterious	Deleterious	Benign	Neutral	0.24	Disease causing
f11‐278	chr3:48680471	G	C	*CELSR3*	c.8335C>G	p.Arg2779Gly	Missense	Deleterious	Deleterious	Benign	Neutral	0.306	Disease causing
465F99	chr4:126372318	G	A	*FAT4*	c.10147G>A	p.Gly3383Ser	Missense	Deleterious	Deleterious	Probably damaging	NA	0.435	Disease causing
229F08	chr4:126384816	C	T	*FAT4*	c.11893C>T	p.Pro3965Ser	Missense	Deleterious	Tolerated	Benign	NA	0.642	Disease causing
00F191	chr4:55156627	CAG	C	*PDGFRA*	c.3029_3030delAG	p.Arg1011ThrfsTer4	Frameshift	NA	NA	NA	NA	NA	Disease causing
f93‐80	chr6:43098128	A	G	*PTK7*	c.655A>G	p.Ser219Gly	Missense	Neutral	Tolerated	Benign	Neutral	0.418	Disease causing
8F97	chr7:17915130	C	A	*SNX13*	c.637G>T	p.Val213Leu	Missense	Neutral	Deleterious	Benign	Deleterious	0.168	Disease causing
88F97*	chr8:144887545	C	T	*SCRIB*	c.2407G>A	p.Ala803Thr	Missense	Neutral	Deleterious	Possibly damaging	NA	0.118	Disease causing
f93‐3	chr8:144890990	T	C	*SCRIB*	c.1904A>G	p.Asp635Gly	Missense	Deleterious	Deleterious	Benign	NA	0.041	Polymorphism
439F03	chr10:82040481	G	T	*MAT1A*	c.360C>A	p.Cys120Ter	Stop‐gained	NA	NA	NA	NA	NA	Disease causing
485F06	chr11:34477676	G	C	*CAT*	c.830G>C	p.Trp277Ser	Missense	Deleterious	Deleterious	Probably damaging	Deleterious	0.908	Disease causing
556F05	chr12:6647018	C	G	*GAPDH*	c.794C>G	p.Ala265Gly	Missense	Neutral	Deleterious	Benign	Neutral	0.33	Disease causing
f94‐114	chr14:37050297	T	C	*NKX2‐8*	c.530A>G	p.Asp177Gly	Missense	Deleterious	Deleterious	Possibly damaging	Deleterious	0.652	Disease causing
99F553	chr15:58285237	A	G	*ALDH1A2*	c.590T>C	p.Ile197Thr	Missense	Deleterious	Deleterious	Probably damaging	Deleterious	0.384	Disease causing
43F05	chr17:26096144	G	T	*NOS2*	c.1893C>A	p.Tyr631Ter	Stop‐gained	NA	NA	NA	NA	NA	Disease causing
55F08	chr17:7577088	T	A	*TP53*	c.850A>T	p.Thr284Ser	Missense	Neutral	Tolerated	Possibly damaging	Deleterious	0.495	Polymorphism
97F91	chr19:45871937	A	C	*ERCC2*	c.311T>G	p.Phe104Cys	Missense	Deleterious	Deleterious	Possibly damaging	Deleterious	0.776	Disease causing
01F373	chr22:31006935	G	C	*TCN2*	c.142G>C	p.Glu48Gln	Missense	Neutral	Deleterious	Benign	Deleterious	0.129	Polymorphism
88F97*	chrX:9859044	C	A	*SHROOM2*	c.345C>A	p.His115Gln	Missense	Deleterious	Deleterious	Possibly damaging	Deleterious	0.171	Disease causing

Abbreviations: A, altered base; NA, not applicable; R, reference base.

Higher REVEL score indicate increased likelihood that the variant is disease‐causing. 88F97* had more than 1 novel damaging variants.

**Figure 2:**
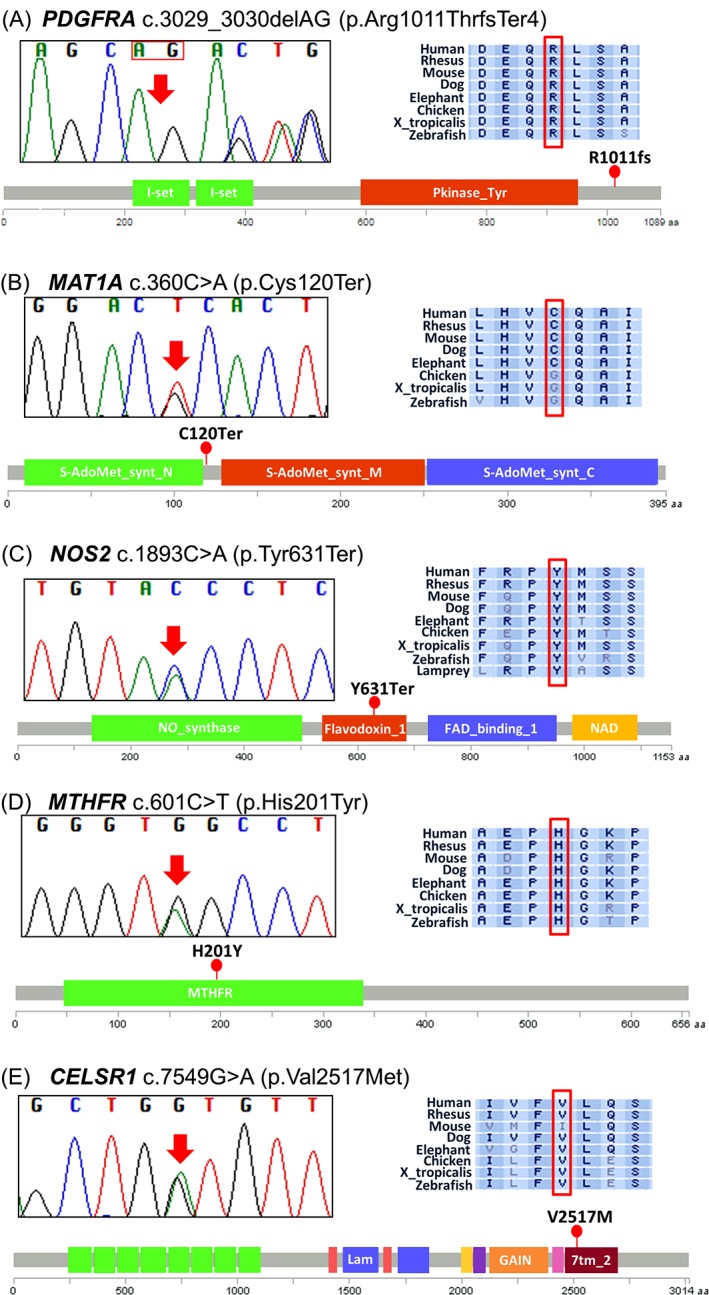
DNA sequence electropherograms, protein conservations and position of the genetic variants in relation to the protein domains. Protein domain figures were created with MutationMapper (http://www.cbioportal.org/mutation_mapper.jsp, v1.0.1). (A) *PDGFRA* frameshift deletion mutation. I‐set, immunoglobulin I‐set domain; Pkinase_Tyr, protein tyrosine kinase. (B) *MAT1A* stop‐gained mutation. S‐AdoMet_synt_N, S‐adenosylmethionine synthetase, N‐terminal domain. S‐AdoMet_synt_M, S‐adenosylmethionine synthetase, central domain; S‐AdoMet_synt_C, S‐adenosylmethionine synthetase, C‐terminal domain. (C) *NOS2* stop‐gained mutation. NO_synthase, nitric oxide synthase, oxygenase domain; Flavodoxin_1, flavodoxin. FAD_binding_1, FAD binding domain; NAD_binding_1, oxidoreductase NAD‐binding domain. (D) *CELSR1* missense mutation. Lam, Laminin G domain; GAIN, GPCR‐autoproteolysis inducing (GAIN) domain; 7tm_2, 7 transmembrane receptors. (E) *MTHFR* missense mutation. MTHFR, methylenetetrahydrofolate reductase

We identified 3 novel loss‐of‐function (LoF) variants. These included a frameshift variant c.3029_3030delAG which introduces a premature stop codon (p.Arg1011ThrfsTer4) in the platelet derived growth factor receptor alpha (*PDGFRA*) gene (Figure 2A). In ExAC, 3 unique LoF variants are reported in *PDGFRA* with a pLI = 1, indicating extreme intolerance for LoF. *Pdgfra*‐null mice are embryonic lethal with severe NTDs and *PDGFRA* promoter haplotypes have been associated with NTD susceptibility in humans.^34^, ^35^ A stop‐gain variant c.360C>A (p.Cys120Ter) was detected in the methionine adenosyltransferase 1A (*MAT1A*) gene (Figure 2B). In ExAC, 2 unique stop‐gain LoF variants are reported in *MAT1A* with a pLI = 0.75. *MAT1A* encodes methionine adenosyltransferase (MAT) which catalyzes the conversion of methionine to S‐adenosylmethionine (SAM), the universal methyl donor. Mutations in the *MAT1A* gene are reported to cause MAT I/III deficiency, a condition characterized by persistent hypermethioninemia not accompanied by elevated homocysteine or tyrosine.^36^ Disturbance in the methionine cycle has been found to cause NTDs in experimental models in the mouse.^31^ However, clinical manifestations of MAT deficiency are variable, including no neurological abnormalities.^36^ Also, a stop‐gain variant c.1893C>A (p.Tyr631Ter) was detected in the nitric oxide synthase 2 (*NOS2*) gene (Figure 2C). In ExAC, 20 LoF variants are reported in *NOS2* with a pLI = 0, suggesting tolerance of LoF and low confidence of causal effect. However, *NOS2* has been previously associated with a cranial NTD phenotype where A/G genotype of the rs4795067 SNP within *NOS2* was shown to be associated specifically with increased cranial NTD risk.^37^

One individual (283F06) was heterozygous for a novel missense variant in the catalytic N‐terminal domain of the methylenetetrahydrofolate reductase (*MTHFR*) gene (c.601C>T; p.His201Tyr) (Figure 2D), which was predicted to be damaging by all 6 mutation predictors tested (Table 1). This individual was also heterozygous for the common *MTHFR* c.677C>T variant, and also carries a rare glycine decarboxylase (*GLDC*) c.2203G>T missense variant, possibly indicating a compromised FOCM in this patient. Interestingly, 2 unrelated patients harbor an identical extremely rare (gnomAD frequency 1/276 358) missense variant (c.7549G>A; p.Val2517Met) within the transmembrane receptor domain of the cadherin, EGF LAG seven‐pass G‐type receptor 1 (*CELSR1*) gene, which encodes a core protein of the PCP pathway (Figure 2E, Table S2 in Appendix S3). Heterozygous missense mutations in *CELSR1* gene have previously been reported in a number of NTD patients.^38^, ^39^, ^40^ Two novel and 3 rare *SCRIB* missense variants were identified in 5 anencephaly cases (Table 1 and Table S2 in Appendix S3). *SCRIB* mutations have previously been implicated in human craniorachischisis.^40^ Three samples carried more than 1 variant within the same gene: sample 01F292 had 2 rare *FAT4* variants (c.739C>A; c.6607C>T), f11‐278 had 1 novel (c.8335C>G) and 1 rare (c.5587C>T) variants in *CELSR3* and 693F06 had 2 rare missense variants (c.3109G>C; c.824G>A) in *NOS2*.

### 3.4 Genes harboring multiple novel and rare variants

In 51/191 genes we identified more than 1 novel and/or rare variants predicted to be damaging (Table S2 in Appendix S3). To assess for potential enrichment of rare/novel damaging variants in the NTD cohort (*N* = 90) compared to the ExAC controls (*N* = 60 706), a mutation burden analysis was carried out. To eliminate the possibility of identifying significant enrichment differences due to sequencing coverage variation between the cohorts, percentage coverage of the targeted regions at ≥30 read depth for all 191 genes were compared between the NTD cases and the ExAC controls. As expected, on average NTD cases showed higher percentage coverage, due to the nature of the targeted capture sequencing design. Therefore, further analysis was limited to genes showing percentage differences not greater than 3.5% between the cohorts. This resulted in 13 genes that were comparable to each other (97.5% in NTD cases vs 95.7% in controls; *t* test *P*‐value = 0.13, 2‐tailed), and 8 of these genes (*COBL*, *FAT4*, *MTRR*, *PDGFRA*, *PRICKLE2*, *SALL4*, *TCN2* and *TXN2*) had at least 1 rare/novel variant predicted to be damaging which could be used for further analysis. Using Fisher's exact test, we identified 4 genes (*COBL*, *FAT4*, *PDGFRA* and *TXN2*) that showed significant enrichment in the NTD cases (Table 2). Of these, *COBL* and *FAT4* stayed statistically significant after the multiple test correction (Table S4 in Appendix S3).

**Table 2:** Mutation burden analysis result

Gene	Cases (*N* = 90)		Controls (*N* = 60 706)		Percentage coverage at ×30 case/control	95% CI	OR	*P*‐val	*P*‐adj
	With variants	Without variants	With variants	Without variants					
*COBL*	5	85	331	60 375	90.6/88.9	3.37‐26.28	10.7	0.00015	**0.00195**
*FAT4*	13	77	1110	59 596	96.8/93.5	4.60‐16.50	9.1	1.2E‐08	**1.56E‐07**
*PDGFRA*	2	88	156	60 550	99.9/97.6	1.04‐33.35	8.8	0.02315	0.30095
*TXN2*	1	89	31	60 675	96.5/98.1	0.53‐134.93	22.0	0.04631	0.60203

Abbreviations: CI, confidence interval; OR, Odds ratio; *P*‐val, nominal *P*‐values; *P*‐adj, Bonferroni corrected *P*‐values.

Mutation burden analysis was performed for the 191 genes in the panel. Genes that showed significant differences are shown. Because sample‐level information is not available at the ExAC database, the burden analysis was performed assuming that each variant serves as an independent sample. To allow unbiased selection, the gene‐level coverage between the NTD cases and ExAC control was compared using Student's *t* test (2‐tailed). The enrichment of the novel/rare variants predicted to be damaging were assessed using Fisher's exact test (2‐tailed).

### 3.5 Multiple damaging variants by samples

Finally, we investigated the possible effects of multiple gene interaction within the same samples. On average, each NTD patient sample carried 9 rare/novel variants, with approximately 3 of these variants predicted to be damaging. In the NTD capture controls (*n* = 4 unrelated), each carried 2 novel/rare variants on average, with approximately 1.5 of these predicted to be damaging (Table S3 in Appendix S3). Out of 90 NTD cases, 75 carried more than 1 novel/rare damaging variants involving 98 candidate genes (Table S2 in Appendix S3). As can be seen from the diversity of the genes affected, there was no clear recurring pattern in which the same set of genes was affected throughout the samples; rather, each case exhibited an individually unique set of variants. However, some of the patients showed multiple hits within PCP‐associated genes. Five anencephaly cases carried rare or novel *CELSR1* missense variants, three of whom carried additional rare potentially damaging PCP variants: 01F377 (*CELSR1* c.6362G>A and *PRICKLE4* c.730C>G), 2F07 (*CELSR1* c.8807C>T and *DVL3* c.1622C>T), 618F05 (*CELSR1* c.8282C>T and *SCRIB* c.3979G>A). One patient (f93‐80) had a novel *PTK7* missense variant (c.655A>G) with a rare *CELSR2* missense variant (c.1892C>T). Three patients carried missense variants both in *FZD* and other PCP‐associated genes: 01F552 (*FZD6* c.1531C>T and *CELSR2* c.3800A>G), 335F07 (*FZD6* c.544G>A and 2 *FAT4* missense variants c.5792A>G; c.10384A>G), and 465F99 (rare *FZD1* missense variant c.211C>T and a novel *FAT4* missense variant c.10147G>A).

## 4 DISCUSSION

In this study we have performed a comprehensive targeted exome sequencing analysis of NTD candidate genes in a cohort of unrelated fetuses with anencephaly and craniorachischisis, to identify genetic variants associated with these disorders. Few genetic studies have comprehensively investigated anencephaly, which may reflect the extra difficulty of collecting samples following termination rather than live born NTD cases, which are usually either spina bifida aperta, or closed spinal dysraphism. We have examined 191 genes previously implicated in either mouse or human NTDs, the majority of which have not been previously analyzed in a large cohort of anencephaly patients or studied for polygenic or burden effects.

### 4.1 *PDGFRA*

A novel *PDGFRA* frameshift variant c.3029_3030delAG leading to premature stop codon (p.Arg1011ThrfsTer4) was identified in an anencephalic patient. *PDGFRA* is a cell‐surface tyrosine kinase receptor, which has an essential role in embryonic development and cell proliferation.^41^ Homozygous *Pdgfra* mutant mice are embryonic lethal presenting with a wavy neural tube and cranial region closure failure.^34^ In humans, the *PDGFRA*‐promoter haplotype H1 was found to confer low transcriptional activity and has been associated with increased NTD risk.^35^ Therefore, it is possible that the reduced abundance of PDGFRA caused by the LoF variant could have led to the anencephaly phenotype in our patient. In addition, 2 other cases (99F590 and 735F97) carried a rare missense variant c.236G>A (p.Gly79Asp) in *PDGFRA*. Overall, these findings resulted in a nominal significant enrichment of *PDGFRA* variants in NTD cases compared to the healthy controls in the burden analysis.

### 4.2 Folate one‐carbon metabolism

Despite the well‐documented benefits of folic acid in NTD prevention, the actual mechanism of folate action is still unknown. Genes involved in folate one‐carbon metabolism (FOCM) have been studied extensively as NTD candidates. Early reports described an association between NTDs and a common variant c.677C>T within the *MTHFR* gene in Dutch and Irish populations, although a subsequent meta‐analysis concluded that the increased NTD risk for this variant was only present within the Irish population (reviewed in Reference 42). Subsequently many genes encoding folate pathway enzymes, transporters and receptors have been studied with mostly inconsistent findings.^7^ More recently, several candidate variants were identified in *AMT* and *GLDC,* 2 of the genes constituting the mitochondrial GCS.^10^, ^32^ In the present study, we identified a novel missense variant affecting the catalytic domain of the *MTHFR* gene. This patient additionally carried the c.677C>T variant, and a rare missense variant (c.2203G>T) in the *GLDC* gene. It is possible that this combination of genotypes caused sub‐optimal folate metabolism, leading to anencephaly. In addition, we have identified 4 more patients (1 craniorachischisis and 3 anencephaly) carrying rare *GLDC* variants predicted to be damaging. One patient (735F97) was heterozygous for the missense variant c.2852C>A (p.Ser951Tyr) previously reported in a patient with NKH.^30^ While *Mthfr*‐null mice do not display a NTD phenotype, *Gldc* deficient mice variably display NTDs and/or features of NKH.^11^ We did not identify variants in other GCS genes, *AMT* or *GCSH*, after filtering for frequency (<1% MAF) and mutation prediction in our cohort.

### 4.3 Gene‐gene interactions and the PCP pathway

Both gene‐environment and gene‐gene interactions are believed to play an important role in NTD etiology. In humans, NTD cases are mostly sporadic and putative mutations reported to date are predominantly heterozygous, sometimes with incomplete penetrance.^8^ Therefore, it has been suggested that human NTDs exhibit a polygenic or oligogenic pattern of inheritance involving multiple heterozygous gene mutations. Such genetic interactions are evident from the many doubly heterozygous NTD mouse models that have been described.^43^

In the present study, the majority of cases (75/90) carried more than 1 rare damaging variant, without revealing an obvious pattern of co‐inheritance. This might be partly attributed to the fact that the analysis was conducted using a MAF <1% and damaging predictions, which might have excluded a possible contribution of more common alleles. Despite this, we identified 3 anencephalic fetuses that were double heterozygotes, carrying missense variants in *CELSR1* gene and a variant in other PCP‐related genes *SCRIB*, *PRICKLE4*, and *DVL3*. In addition, 2 unrelated anencephalic patients carried the same, extremely rare *CELSR1* missense variant (c.7549G>A; 1/276 358 gnomAD). Rare variants in *CELSR1* have previously been reported in both open and closed NTD types.^38^, ^39^, ^40^ Two previous NTD cases have been reported to be doubly heterozygous either for *CELSR1* 38 or *DVL2*.^44^ *Celsr1* homozygous mutant (*Celsr1* ^*Crsh*/*Crsh*^) mice show craniorachischisis,^9^ suggesting that the *CELSR1* gene is likely to be implicated in both human and mouse NTD etiology.

### 4.4 Burden analysis

To maximize the number of the cases to be analyzed, only a small number of controls were included in the sequencing platform. Therefore, to perform burden analysis, the public database (ExAC) was used. However, because of the lack of similarity in the sequence coverage in many of the genes between the NTD and control cohorts, we were only able to assess 13/191 genes. *COBL* encodes an actin regulator protein, and the *Vangl2* ^*Lp*^/*Cobl* ^*C101/C101*^ mutant mice show exencephaly, indicating an interaction between these genes.^45^ *FAT4* encodes a protocadherin family protein that is associated with the PCP pathway.^46^ A study of *Fat4*‐null mice described impaired convergent extension with faulty ureteric tubule elongation.^46^ However, in humans, mutations in this gene are implicated in both Van Maldergem Syndrome 2 [OMIM: 615546], characterized by intellectual disability with typical craniofacial features, and Hennekam Lymphangiectasia‐Lymphedema Syndrome 2 [OMIM: 616006], which causes lymphatic dysplasia.

### 4.5 Study limitations

No parental samples were available for this study, excluding the possibility of identifying de novo mutations or evaluating penetrance or co‐inheritance of risk alleles from each parent. Due to the lethality of this phenotype, it is also difficult to ascertain familial anencephalic cases. Only DNA was available which excluded the possibility of testing gene/protein expression associations with the variants. As a candidate gene approach, we were unable to comprehensively include all genes that could be implicated in NTD causation. This can more easily be achieved using whole exome/genome sequencing (WES/WGS) in a hypothesis‐free manner. However, recent WES studies^32^, ^47^ have still focused on analysis of known candidate genes. Our data and findings of these other studies concur that no major recurring genetic defect explains any substantial group of NTDs, consistent with the notion that all NTD types are most likely complex traits involving interaction of multiple loci and non‐genetic factors. It remains possible that substantial genetic contributions come from outside the coding regions, which would also be missed in WES. Future investigations will need to include non‐coding regulatory regions (eg, enhancers), using WES for more comprehensive analysis. However, identification of causal variants can be even more challenging at this level. For the moment the authenticity of reported human NTD mutations still needs to be unequivocally verified. Although functional analysis was beyond the scope of this study, it would be of great benefit to test candidate variants in mouse models to provide confirmation of causal effect.

## 5 CONCLUSION AND FUTURE STUDIES

We have identified novel and rare variants within genes with known biological association with NTDs, specifically focusing on folate metabolism and PCP pathways. This study highlights the potential involvement of PCP genes including *CELSR1* in association with anencephaly phenotypes, and also *PDGFRA* as strong NTD candidates in humans. Further identification and functional testing of genetic factors will lead to improved understanding of molecular mechanisms of NTD, and ultimately may help to create a targeted and cost‐effective method of screening genes for the clinical management of families with a history of NTD‐affected pregnancy.

## Supporting information


**Appendix S1.**

**Appendix S2.**

**Appendix S3.**
