# Supplementary material for: A targeted sequencing panel identifies rare damaging variants in multiple genes in the cranial neural tube defect, anencephaly
Source: Clin Genet. 2018 Feb 11;93(4):870–9. doi: 10.1111/cge.13189 (PMC5887939; doi:10.1111/cge.13189)
Supplement: Supplementary file 1 — Appendix S1. [file CGE-93-870-s001.pdf]

# SUPPLEMENTARY FIGURES

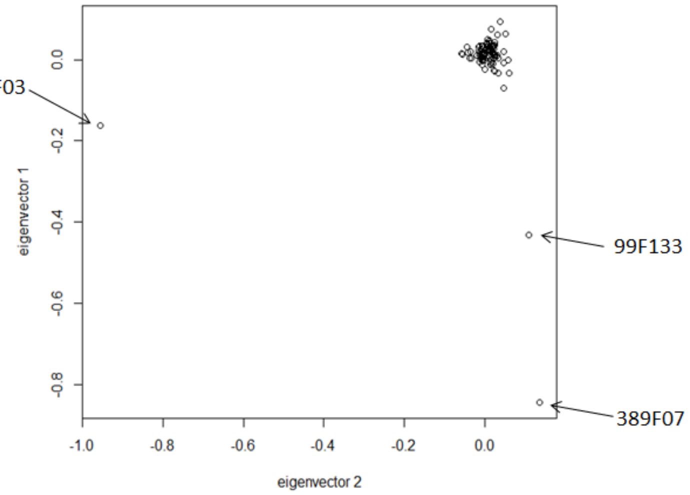


**Supplementary Figure S1. Principle Component Analysis of NTD samples.**

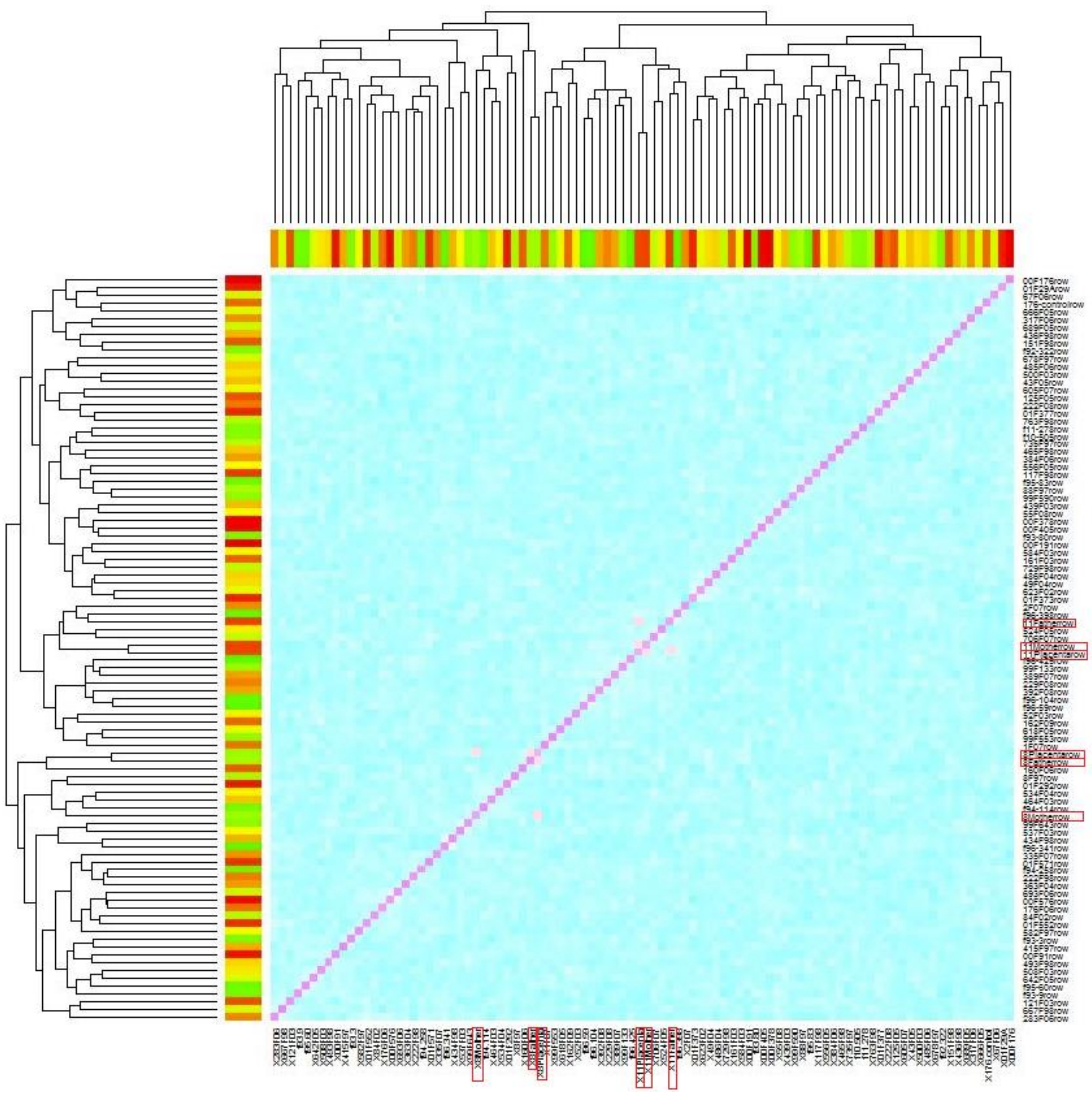

**Supplementary Figure S2. Relatedness analysis of the samples used in the capture sequences.** 100% identical samples are indicated as the strong purple squares, whereas blue squares represent unrelated samples. Each sample was compared with all the other samples including themselves (presented as the purple line across the heatmap). Only healthy control trio samples (child-mother-father, in red rectangles) showed relatedness, indicated as pale pink squares.
