# Supplementary material for: A targeted sequencing panel identifies rare damaging variants in multiple genes in the cranial neural tube defect, anencephaly
Source: Clin Genet. 2018 Feb 11;93(4):870–9. doi: 10.1111/cge.13189 (PMC5887939; doi:10.1111/cge.13189)
Supplement: Supplementary file 2 — Appendix S2. [file CGE-93-870-s002.pdf]

Supplementary Methods

Principal component analysis (PCA) and relatedness test of SNP data

Since the family history and ethnicity of the NTD samples were not available, sample relatedness was assessed using the shared rare (MAF <5%) single nucleotide variant (SNV) genotype calls. The R statistical package ‘SNPrelate’ (v1.8.0) was used for principal component analysis (PCA). RVTESTS[1] was used to calculate autosomal kinship matrix. A heatmap was generated with R (v3.2.2).

Genetic variant validation

Previously unreported (novel) genetic variants identified in the capture sequencing were verified by Sanger sequencing (primer sequences provided in a table below). Polymerase chain reaction (PCR) was carried out with BIOTAQ DNA Polymerase (BIOLINE, London, UK). PCR products were purified by microclean (microzone, Haywards Heath , UK) and amplified with BigDyeTerminator v1.1, followed by analysis on a DNA Analyzer 3070 (Applied Biosystems, California, US). The resulting read-outs were analysed by Sequencher (v5.3: Gene Codes).

**Genes**	**Sequence (5’ > 3’)**	**RefSeq**
MTHFR_F	CCTCTCCTGACTGTCATCCC	NM_005957
MTHFR_R	ACTCAGCGAACTCAGCACTC	
NID1_F	CCCAGAATCCTAGGTTCTTGC	NM_002508
NID1_R	TCCCACTGAACCTTGACTGA	
ALDH1L1_F	CCACACCTGACTTGGAGGAC	NM_001270364
ALDH1L1_R	ACTGTGTAGGTACCTGGCCT	
CELSR3_F	CACCGACCTTACAGACTCCA	NM_001407

CELSR3_R	GCTAACCCTGAAGCAGCCTA	
FAT4_F1	TTGCTAGCGACCGTGATTTG	NM_001291285
FAT4_R1	CTGGGGATGGAGTCTGAGTC	
FAT4_F2	TGGTAATGGTGTAACGGTGTT	
FAT4_R2	CCAATGCAATGTATTCAATGGGA	
PDGFRA_F	ACTTCCTGAAGAGTGACCATCC	NM_001347828
PDGFRA_R	TTATCGGGAAAATGGGTCCTCTC	
PTK7_F	TCAGAGCAACCACACAGTCA	NR_072998
PTK7_R	CACCCTGGCAAAGCTTTCAT	
SNX13_F	CGAGTATTCAGAAAGGCTCAACA	NM_015132
SNX13_R	GCTAACTTGACTCTAGCCAGC	
SCRIB_F1	AAGGTCTCGGGTGATGTCAG	NM_015356
SCRIB_R1	TGTAATCATCCTCGGGCCG	
SCRIB_F2	TCAAGATCTCCAAGCTGCCC	
SCRIB_R2	GAGGCTGACACCAACCTTGA	
MAT1A_F	GTGGCTTTGGAGCAGCAATC	NM_000429
MAT1A_R	CAACTCGCTCCTGGCTATCG	
CAT_F	TTTCTGTTGAAGATGCGGCG	NM_001752
CAT_R	TGCTGGTAAGCACTCATTCAC	
GAPDH_F	AACGTGTCAGTGGTGGACCT	NM_001256799
GAPDH_R	CCTGCACTTTTTAAGAGCCAGTC	
NKX2-8_F	GGTTCCAGAATCATCGCTACA	NM_014360
NKX2-8_R	CTCACCAGTTCCAGGAGACC	
ALDH1A2_F	AGAGATTTCCAAGGTCAGTGCT	NM_003888

ALDH1A2_R	ACGTGGCTGATGAAAGCTGA	
NOS2_F	GTGGTCATGGGAGTTGGTGA	NM_000625
NOS2_R	CACTGAGCTCATCCCCTTCT	
TP53_F	AAAGGACAAGGGTGGTTGGG	NM_000546
TP53_R	TACCTCGCTTAGTGCTCCCT	
ERCC2_F	TTTGGGAAGCTGGGGAAGAG	NM_000400
ERCC2_R	GAGATATGGCCTGGGGATCC	
TCN2_F	AAATACCAGAGATGGACAGCCA	NM_001184726
TCN2_R	AGTACCCAAGTTTCCCATCTCTTAG	
SHROOM2_F	CAAGCCCCCTGCACTGTTT	NM_001649
SHROOM2_R	AGGCTGAACAAACCTGGCAT	

*An annealing temperature for all primers is 58 °C.

Sanger sequencing validation was additionally carried out with the following list of primers for the previously reported rare damaging variants within *COBL* and *FAT4*, which were used for the mutation burden analysis.

**Genes**	**Sequence (5' > 3')**	**RefSeq**
FAT4_F6	TCGCAAGTGGGAATCTTGGC	NM_001291285
FAT4_R6	TGTCCTTGTCCATTGCCGAA	
FAT4_F8	GTTCCTGCATCTGGTGTCCA	
FAT4_R8	TAAGTCCCGTCTCAGGGTCC	
FAT4_F9	GGCACAAATGGACAGGTTCG	
FAT4_R9	CCAGTAGAACAATGCTGACCG	
FAT4_F10	GGAACCCCTTAGTGCTACTGT	

FAT4_R10	TCCGCCTCATAAGAGACTCCC	
FAT4_F11	CTGTTCGAGCAGAAGATGGTG	
FAT4_R11	TTCTATTCCTCAACTTCCAACCT	
FAT4_F12	ACCGTGCTTGATGCAAATGAC	
FAT4_R12	CAGCATGGGCCCGTTATCAT	
FAT4_F13	ACTGGGACTAACAACCACGGA	
FAT4_R13	GTCTGCATCAGGGTCATGTG	
FAT4_F14	TTTGGCATCTCCAGAGCAGG	
FAT4_R14	GACAGAGGGTTGGGAGTTGG	
FAT4_F15	TTCAGTCTGAGCACTGCTGG	
FAT4_R15	TCAAACGTGCCATCTGTGGA	
FAT4_F16	CTGCGAGTTGAACAGTTATGGA	
FAT4_R16	CTGACACCTTCTTCATGGTGGT	
FAT4_F17	GCAACACCTGCCTATTCCCTT	
FAT4_R17	TTCCGTGGTTGTTAGTCCCA	
COBL_F1	GTGTGGGAGGACCTGGAAAG	NM_001287436
COBL_R1	CACTCACCCATCGTGCTCTT	
COBL_F3	TGGCCCCAGGCATATTATGTA	
COBL_R3	ACCCCGAATCCACAGGATCA	
COBL_F4	GGCCGACAGACTGATGTCAC	
COBL_R4	TGCGAGTCCAGAGAGACGAT	
COBL_F5	GCCCACACAGGAAGTTGTCT	
COBL_R5	ACAGAGCCATTGACCAGCTC	
COBL_F6	TCTTCCCAGAGGGATCGTGT	

COBL_R6	TGGGACTGGTCTCTGGACAA	

*An annealing temperature for all primers is 58 °C.

REFERENCE

1. Zhan X, Hu Y, Li B, Abecasis GR, Liu DJ. RVTESTS: an efficient and comprehensive tool for rare variant association analysis using sequence data. *Bioinformatics* 2016;32(9):1423-6. doi: 10.1093/bioinformatics/btw079
