# Supplementary material for: A targeted sequencing panel identifies rare damaging variants in multiple genes in the cranial neural tube defect, anencephaly
Source: Clin Genet. 2018 Feb 11;93(4):870–9. doi: 10.1111/cge.13189 (PMC5887939; doi:10.1111/cge.13189)
Supplement: Supplementary file 3 — Appendix S3. [file CGE-93-870-s003.pdf]

**Supplementary Table S1. List of genes inlcuded in the capture sequencing.**

| Gene symbol | Gene name | Locus | Pathway/Function (RefSeq Summary) | Relevance to NTD | Refs |
|---|---|---|---|---|---|
| ***AHCY*** | adenosylhomocysteinase | 20q11.22 | Hydrolyses S-adenosylhomocysteine to L-homocysteine and adenosine. | Folate one-carbon metabolism. | 1 |
| ***ALDH1A2*** | aldehyde dehydrogenase 1 family member A2 | 15q21.3 | Catalyses the retinoic acid synthesis from retinaldehyde. | Retinol metabolism. Previously associated with Spina NTD risk in a USA cohort. | 2 |
| ***ALDH1L1*** | aldehyde dehydrogenase 1 family member L1 | 3q21.3 | Catalyses the conversion of nicotinamide adenine dinucleotide phosphate (NADP+), 10-formyltetrahydrofolate, and water to NADPH, tetrahydrofolate, and carbon dioxide. | Folate one-carbon metabolism. Previously associated with NTD in a Han Chinese cohort and a nominal significance in a Dutch cohort. | 3, 4 |
| ***ALX1*** | Aristaless-like homeobox 1 (Also known as CART1) | 12q21.31 | Involved in forebrain mesenchyme formation in rodents. | NTDs in mouse mutant. | 5 |
| ***AMBRA1*** | autophagy and beclin 1 regulator 1 | 11p11.2 | Regulates autophagosome formation. | NTDs in mouse mutant. | 6 |
| ***AMD1*** | adenosylmethionine decarboxylase 1 | 6q21 | Intermediate enzyme in polyamine biosynthesis. | Folate one-carbon metabolism. . | 1 |
| ***AMT*** | aminomethyltransferase | 3p21.31 | Glycine cleavage multienzyme system constituent. | Folate one-carbon metabolism. NTDs in mouse mutant. | 1,7 |
| ***APEX1*** | apurinic/apyrimidinic endodeoxyribonuclease 1 | 14q11.2 | DNA repair enzyme. | Previously associated with NTD in a USA cohort. | 8 |
| ***ARID1A*** | AT-rich interaction domain 1A | 1p36.11 | ATP-dependent chromatin remodeling complex SNF/SWI constituent. | NTDs in mouse mutant. | 9 |
| ***BBS4*** | Bardet-Biedl syndrome 4 | 15q24.1 | Part of a protein complex required for ciliogenesis. | NTDs in mouse mutant. | 10 |
| ***BHMT*** | betaine--homocysteine S-methyltransferase | 5q14.1 | Catalyses the conversion of homocysteine and betaine to methionine and dimethylglycine. | Folate one-carbon metabolism. Conflicting evidence of association with NTD risk in multiple populations. | 1,11-13 |
| ***BHMT2*** | BHMT2 betaine--homocysteine S-methyltransferase 2 | 5q14.1 | Catalyse the methyl group transfer from betaine to homocysteine. | Folate one-carbon metabolism. | 1 |
| ***BIRC5*** | baculoviral IAP repeat containing 5 | 17q25.3 | Inhibitor of apoptosis (IAP). | NTDs in mouse mutant. | 14 |
| ***BMP2*** | bone morphogenetic protein 2 | 20p12.3 | Bone and cartilage development. | NTDs in mouse mutant. | 15 |
| ***BMP4*** | bone morphogenetic protein 4 | 14q22.2 | Heart development and adipogenesis regulation. | NTDs in mouse mutant. | 16 |
| ***BRCA1*** | BRCA1, DNA repair associated | 17q21.31 | Tumor suppressor and a role in genome stability. | NTDs in mouse mutant. | 17 |
| ***BRD2*** | bromodomain containing 2 | 6p21.32 | BET (bromodomains and extra terminal domain) family transcriptional regulator. | NTDs in mouse mutant. | 18 |
| ***CAT*** | catalase | 11p13 | Antioxidant enzyme. | Oxidative stress. Lower concentration of CAT reported in NTD cases. | 19 |
| ***CBS*** | cystathionine-beta-synthase | 21q22.3 | Catalyse the conversion of homocysteine to cystathionine. | Folate one-carbon metabolism pathway. | 1 |
| ***CELSR1*** | cadherin EGF LAG seven-pass G-type receptor 1 | 22q13.31 | Receptor-ligand interactions, cell adhesion and embryogenesis. | NTDs in mouse mutant. Potentially damaging variants reported in various forms of human NTDs. Planar cell polarity (PCP) pathway. | 20-22 |
| ***CELSR2*** | cadherin EGF LAG seven-pass G-type receptor 2 | 1p13.3 | Involved in facial branchiomotor (FBM) neurons migration. | Planar cell polarity (PCP) pathway. | 23 |
| ***CELSR3*** | cadherin EGF LAG seven-pass G-type receptor 3 | 3p21.31 | Possibly involved in fibre tract formation. | Planar cell polarity (PCP) pathway. | 23 |
| ***CFL1*** | cofilin 1 | 11q13.1 | Intracellular actin-modulating protein. | NTDs in mouse mutant. Previously associated with NTD risk in a USA cohort. | 24,25 |
| ***CHKA*** | choline kinase alpha | 11q13.2 | Catalyses the ethanolamine phosphorylation. | Folate one-carbon metabolism. Previously associated with NTD risk in a USA cohort. | 26 |
| ***CITED2*** | Cbp/p300 interacting transactivator with Glu/Asp rich carboxy-terminal domain 2 | 6q24.1 | Transactivation inhibition of HIF1A-induced genes. | NTDs in mouse mutant. | 27 |
| ***COBL*** | cordon-bleu WH2 repeat protein | 7p12.1 | Actin regulator. | NTDs in mouse mutant. | 28 |
| ***COQ3*** | coenzyme Q3, methyltransferase | 6q16.2 | Methylates coenzyme Q intermediates to convert to coenzyme Q. | Methylating agent. | 1 |
| ***CRABP1*** | cellular retinoic acid binding protein 1 | 15q25.1 | Involved in retinoic acid-mediated differentiation and proliferation. | Retinol metabolism. | 1 |
| ***CRABP2*** | cellular retinoic acid binding protein 2 | 1q23.1 | Role in retinoid signaling pathway. | Retinol metabolism. | 1 |
| ***CTH*** | cystathionine gamma-lyase | 1p31.1 | Converts cystathione to cysteine. | Folate one-carbon metabolism. | 1 |
| ***CTHRC1*** | collagen triple helix repeat containing 1 | 8q22.3 | Possible Wnt cofactor and a role in vascular remodeling. | PCP pathway. NTDs in mouse mutant (double heterozygous). | 29 |
| ***CUBN*** | cubilin | 10p13 | Receptor for intrinsic factor (vitamin B12 complexes). | Previously associated with NTD risk in a Dutch cohort. | 3 |
| ***CXCL6*** | C-X-C motif chemokine ligand 6 | 4q13.3 | Role in folate metabolism. | Folate one-carbon metabolism. | 30 |
| ***CYP26A1*** | cytochrome P450 family 26 subfamily A member 1 | 10q23.33 | Catalyse drug metabolism. | Retinol metabolism. | 1 |
| ***CYP26B1*** | cytochrome P450 family 26 subfamily B member 1 | 2p13.2 | Catalyse drug metabolism. | Retinol metabolism. | 1 |
| ***DAAM1*** | dishevelled associated activator of morphogenesis 1 | 14q23.1 | Scaffolding protein for the Wnt-induced disheveled-Rho complexa ssembly. | PCP pathway. | 31 |

| | | | | | |
|---|---|---|---|---|---|
| ***DACT1*** | dishevelled binding antagonist of beta catenin 1 | 14q23.1 | Regulates dishevelled-mediated signaling pathways. | PCP pathway. NTDs in mouse mutant. Genetic variants reported in human NTD patients. | 32,33 |
| ***DCHS1*** | dachsous cadherin-related 1 | 11p15.4 | Calcium-dependent cell-cell adhesion molecules. | PCP pathway. | 34 |
| ***DHFR*** | dihydrofolate reductase | 5q14.1 | Converts dihydrofolate into tetrahydrofolate. | Folate one-carbon metabolism. | 1 |
| ***DLC1*** | DLC1 Rho GTPase activating protein | 8p22 | Tumor suppressor gene. | NTDs in mouse mutant. | 35 |
| ***DLG1*** | discs large MAGUK scaffold protein 1 | 3q29 | Scaffolding protein. | PCP pathway. | 36 |
| ***DNMT1*** | DNA methyltransferase 1 | 19p13.2 | Transfers methyl group to cytosine residues which will form 5-methylcytosine. | NTDs in mouse mutant. DNA methylation regulation. | 37 |
| ***DVL1*** | dishevelled segment polarity protein 1 | 1p36.33 | Cytoplasmic phosphoprotein. | PCP pathway. NTDs in mouse mutant (double heterozygous). | 38 |
| ***DVL2*** | dishevelled segment polarity protein 2 | 17p13.1 | Role in signal transduction pathway. | PCP pathway. NTDs in mouse mutant. | 38 |
| ***DVL3*** | dishevelled segment polarity protein 3 | 3q27.1 | Cytoplasmic phosphoprotein. | PCP pathway. NTDs in mouse mutant. | 38 |
| ***EPCAM*** | epithelial cell adhesion molecule | 2p21 | Cell adhesion molecule. | Down-regulated in the *Grhl2* mutant mouse. | 39 |
| ***ERCC2*** | ERCC excision repair 2, TFIIH core complex helicase subunit | 19q13.32 | Nucleotide excision repair. | DNA repair gene. | 40 |
| ***FAT4*** | FAT atypical cadherin 4 | 4q28.1 | Protocadherin family member. | PCP pathway. | 41 |
| ***FJX1*** | four jointed box 1 | 11p13 | Role in growth and differentiation. | PCP pathway. | 42 |
| ***FOLR1*** | folate receptor 1 | 11q13.4 | Bind folic acid and transport 5-methyltetrahydrofolate into cells | Folate one-carbon metabolism. | 43 |
| ***FOLR2*** | folate receptor beta | 11q13.4 | Bind folic acid and transport 5-methyltetrahydrofolate into cells | Folate one-carbon metabolism. Previously associated with NTD risk in a USA cohort. | 43,44 |
| ***FOLR3*** | folate receptor 3 | 11q13.4 | Bind folic acid and transport 5-methyltetrahydrofolate into cells | Folate one-carbon metabolism. Previously associated with NTD risk in a USA cohort. | 44 |
| ***IZUMO1R (FOLR4)*** | IZUMO1 receptor, JUNO | 11q21 | Significant similarity to folate receptors but plays a role in fertility. | Folate one-carbon metabolism? | 45 |
| ***FPGS*** | folylpolyglutamate synthase | 9q34.11 | Maintains folylpolyglutamate concentrations in cytosol and mitochondria. | Folate one-carbon metabolism. | 46 |
| ***FTCD*** | formimidoyltransferase cyclodeaminase | 21q22.3 | Channels 1-carbon units from formiminoglutamate. | Folate one-carbon metabolism. | 1 |
| ***FUZ*** | fuzzy planar cell polarity protein | 19q13.33 | Role in ciliogenesis. | PCP pathway. NTDs in mouse mutant. | 47 |
| ***FZD1*** | frizzled class receptor 1 | 7q21.13 | 7-transmembrane domain proteins receptors for Wnt signaling proteins. | PCP pathway. NTDs in mouse mutant (double heterozygous). | 48 |
| ***FZD2*** | frizzled class receptor 2 | 17q21.31 | 7-transmembrane domain proteins receptors for Wnt signaling proteins. | PCP pathway. NTDs in mouse mutant (double heterozygous). | 48 |
| ***FZD3*** | frizzled class receptor 3 | 8p21.1 | 7-transmembrane domain proteins receptors for Wnt signaling proteins. Core vertebrate PCP gene. | PCP pathway. NTDs in mouse mutant (double heterozygous). | 49 |
| ***FZD4*** | frizzled class receptor 4 | 11q14.2 | 7-transmembrane domain proteins receptors for Wnt signaling proteins. | PCP pathway. | 50 |
| ***FZD5*** | frizzled class receptor 5 | 2q33.3 | 7-transmembrane domain proteins receptors for Wnt signaling proteins. | PCP pathway. | 50 |
| ***FZD6*** | frizzled class receptor 6 | 8q22.3 | 7-transmembrane domain proteins receptors for Wnt signaling proteins. Core vertebrate PCP gene. | PCP pathway. NTDs in mouse mutant (double heterozygous). | 49 |
| ***GAMT*** | guanidinoacetate N-methyltransferase | 19p13.3 | Methyltransferase that converts guanidoacetate to creatine | Folate one-carbon metabolism. | 1 |
| ***GAPDH*** | glyceraldehyde-3-phosphate dehydrogenase | 12p13.31 | Reversible oxidative phosphorylation of glyceraldehyde-3-phosphate | Glucose metabolism. | 1 |
| ***GART*** | phosphoribosylglycinamide formyltransferase, phosphoribosylglycinamide synthetase, phosphoribosylaminoimidazole synthetase | 21q22.11 | De novo purine biosynthesis. | Purine biosynthesis. Folate one-carbon metabolism. | 1 |
| ***GCSH*** | glycine cleavage system protein H | 16q23.2 | Transfers the methylamine group of glycine from the P protein to the T protein | Folate one-carbon metabolism. Glycine cleavage system. | 7 |
| ***GGH*** | gamma-glutamyl hydrolase | 8q12.3 | Catalyses the hydrolysis of folylpoly-gamma-glutamates and antifolylpoly-gamma-glutamates | Folate one-carbon metabolism. | 1 |
| ***GLDC*** | glycine decarboxylase | 9p24.1 | Binds to glycine to transfer methylamine group to the T protein from glycine | Folate one-carbon metabolism. Glycine cleavage system. NTDs in mouse mutant. | 51 |
| ***GPC5*** | glypican 5 | 13q31.3 | Cell surface proteoglycans | Previously associated with NTD risk in USA cohorts. | 52 |
| ***ADGRA3 (GPR125)*** | adhesion G protein-coupled receptor A3 | 4p15.2 | G protein-coupled receptor superfamily. Modulates Dishevelled distribution. | PCP pathway. | 53 |
| ***GRHL1*** | grainyhead like transcription factor 1 | 2p25.1 | Transcription factor. | Member of the grainyhead family. | 54 |
| ***GRHL2*** | grainyhead like transcription factor 2 | 8q22.3 | Transcription factor. | NTDs in mouse mutant. | 39 |

| | | | | | |
|---|---|---|---|---|---|
| ***GRHL3*** | grainyhead like transcription factor 3 | 1p36.11 | Transcription factor. | NTDs in mouse mutant. | 55 |
| ***HK1*** | hexokinase 1 | 10q22.1 | Phosphorylate glucose to produce glucose-6-phosphate | Glucose metabolism. Previously associated with NTD risk in a USA cohort. | 56 |
| ***HK2*** | hexokinase 2 | 2p12 | Phosphorylate glucose to produce glucose-6-phosphate | Glucose metabolism. | 56 |
| ***ICMT*** | isoprenylcysteine carboxyl methyltransferase | 1p36.31 | Posttranslational modification of isoprenylated C-terminal cysteine residues. | Protein methylation. | 1 |
| ***INPP5E*** | inositol polyphosphate-5-phosphatase E | 9q34.3 | Intracellular calcium mobilisation and a second messenger mediating cell response to stimuli. | NTDs in mouse mutant. | 57 |
| ***INS*** | insulin | 11p15.5 | Binds to insulin receptor to stimulate glucose uptake | Glucose metabolism. | 1 |
| ***INSR*** | insulin receptor | 19p13.2 | Binds to insulin or other ligands to activate insulin signalling pathway | Glucose metabolism. | 1 |
| ***INTU*** | inturned planar cell polarity protein | 4q28.1 | Role in regulation of ciliogenesis in Hedgehog-responsive cells | PCP pathway. | 58 |
| ***INVS*** | inversin | 9q31.1 | Part of a complex of ciliary proteins | PCP pathway. | 50 |
| ***ITPK1*** | inositol-tetrakisphosphate 1-kinase | 14q32.12 | Regulates the synthesis of inositol tetraphosphate, inositol pentakisphosphate and inositol hexakisphosphate. | NTDs in mouse mutant. | 59 |
| ***JARID2*** | jumonji and AT-rich interaction domain containing 2 | 6p22.3 | Transcriptional repressor | NTDs in mouse mutant. | 60 |
| ***LAMC1*** | laminin subunit gamma 1 | 1q25.3 | Extracellular matrix glycoproteins | Involved in basement membrane organisation. | 61 |
| ***LEP*** | leptin | 7q32.1 | Regulates energy homeostasis | Glucose metabolism. | 1 |
| ***LEPR*** | leptin receptor | 1p31.3 | Regulates energy homeostasis | Glucose metabolism. | 1 |
| ***LMNB1*** | lamin B1 | 5q23.2 | Component of the nuclear lamina | Previously associated with NTD risk in a study using UK, Swedish and USA cohorts. | 62 |
| ***LRP6*** | LDL receptor related protein 6 | 12p13.2 | Receptor and a co-receptor with Frizzled for Wnt for the canonical Wnt/beta-catenin signaling cascade. | NTDs in mouse mutant. | 20 |
| ***MARCKSL1*** | MARCKS like 1 | 1p35.1 | Role in a formation of adherens junction. | NTDs in mouse mutant. | 63 |
| ***MAT1A*** | methionine adenosyltransferase 1A | 10q22.3 | Catalyses the transfer of the adenosyl moiety of ATP to methionine. | Folate one-carbon metabolism. | 1 |
| ***MAT2A*** | methionine adenosyltransferase 2A | 2p11.2 | Catalyses the production of S-adenosylmethionine from methionine and ATP. | Folate one-carbon metabolism. | 1 |
| ***MED12*** | mediator complex subunit 12 | Xq13.1 | Component of this preinitiation complex | NTDs in mouse mutant. | 64 |
| ***MEN1*** | menin 1 | 11q13.1 | Putative tumor suppressor | NTDs in mouse mutant. | 65 |
| ***MGMT*** | O-6-methylguanine-DNA methyltransferase | 10q26.3 | DNA repair protein | Folate one-carbon metabolism. DNA methylation. | 1 |
| ***MTHFD1*** | methylenetetrahydrofolate dehydrogenase, cyclohydrolase and formyltetrahydrofolate synthetase 1 | 14q23.3 | Interconversion of 1-carbon derivatives of tetrahydrofolate | Folate one-carbon metabolism. Previously associated with NTD risk in several studies. | 1 |
| ***MTHFD1L*** | methylenetetrahydrofolate dehydrogenase (NADP+ dependent) 1 like | 6q25.1 | Synthesis of tetrahydrofolate (THF) in the mitochondrion | Folate one-carbon metabolism. NTDs in mouse mutant. | 66 |
| ***MTHFD2*** | methylenetetrahydrofolate dehydrogenase (NADP+ dependent) 2, methenyltetrahydrofolate cyclohydrolase | 2p13.1 | Nuclear-encoded mitochondrial bifunctional enzyme with methenyltetrahydrofolate cyclohydrolase and methylenetetrahydrofolate dehydrogenaseactivities. | Folate one-carbon metabolism. | 1 |
| ***MTHFD2L*** | methylenetetrahydrofolate dehydrogenase (NADP+ dependent) 2 like | 4q13.3 | Enzyme of adult mitochondria with a role in the formate synthesis pathway. | Folate one-carbon metabolism. | 1 |
| ***MTHFR*** | methylenetetrahydrofolate reductase | 1p36.22 | Catalyses the conversion of 5,10-methylenetetrahydrofolate to 5-methyltetrahydrofolate. | Folate one-carbon metabolism. Previously associated with NTD risk in several studies. | 1 |
| ***MTHFS*** | methenyltetrahydrofolate synthetase | 15q25.1 | Catalyses the conversion of 5-formyltetrahydrofolate to 5,10-methenyltetrahydrofolate | Folate one-carbon metabolism. | 1 |
| ***MTRR*** | 5-methyltetrahydrofolate-homocysteine methyltransferase reductase | 5p15.31 | Synthesis of methionine by regenerating methionine synthase to a functional state. | Folate one-carbon metabolism. Previously associated with NTD risk in several studies. | 1 |
| ***MUT*** | methylmalonyl-CoA mutase | 6p12.3 | Mitochondrial enzyme methylmalonyl Coenzyme A mutase. | Folate one-carbon metabolism. | 1 |
| ***NAT1*** | N-acetyltransferase 1 | 8p22 | Catgalyse the acetyl group transfer from acetyl-CoA to various arylamine and hydrazine substrates | Folate one-carbon metabolism. Acetylation reactions. Previous association with NTD risk in a USA cohort. | 67 |
| ***NAT2*** | N-acetyltransferase 2 | 8p22 | Activate and deactivate carcinogens and hydrazine and arylamine drugs | Folate one-carbon metabolism. Acetylation reactions. | 1 |
| ***NCAM1*** | neural cell adhesion molecule 1 | 11q23.2 | Role in cell-to-cell interactions and cell-matrix interactions during development and differentiation | Cell adhesion. Previously associated with a NTD risk in a USA cohort. | 68 |
| ***NDST1*** | N-deacetylase and N-sulfotransferase 1 | 5q33.1 | Role in heparan sulfate biosynthesis | NTDs in mouse mutant. | 69 |

| | | | | | |
|---|---|---|---|---|---|
| ***NID1*** | nidogen 1 | 1q42.3 | Basement membrane glycoproteins | Basement membrane component. | 70 |
| ***NKX2-8*** | NK2 homeobox 8 | 14q13.3 | Developmental regulator with homeobox domain | Previously associated with NTD risk in a canine GWAS and in a human study in a USA cohort. | 71 |
| ***NNMT*** | nicotinamide N-methyltransferase | 11q23.2 | Drug metabolism by the liver by N-methylation. | Methylation reactions. | 1 |
| ***NOG*** | noggin | 17q22 | Binds and inactivates members of the transforming growth factor-beta superfamily signaling proteins. | NTDs in mouse mutant. | 72 |
| ***NOS1*** | nitric oxide synthase 1 | 12q24.22 | Nitric oxide synthases | Possible effect on folate one-carbon metabolism. | 1 |
| ***NOS2*** | nitric oxide synthase 2 | 17q11.2 | Nitric oxide synthases | Possible effect on folate one-carbon metabolism. | 1 |
| ***NOS3*** | nitric oxide synthase 3 | 7q36.1 | Nitric oxide synthases | Possible effect on folate one-carbon metabolism. | 1 |
| ***PAX3*** | paired box 3 | 2q36.1 | Transcription factor | NTDs in mouse mutant. | 73 |
| ***PCMT1*** | protein-L-isoaspartate (D-aspartate) O-methyltransferase | 6q25.1 | Protein carboxyl methyltransferase enzyme with a role in protein repair. | Previous association with NTD risk in several human cohort studies. | 1,74 |
| ***PCYT1A*** | phosphate cytidylyltransferase 1, choline, alpha | 3q29 | Regulation of phosphatidylcholine biosynthesis | Previous association with NTD risk in a USA cohort study. | 26 |
| ***PDGFRA*** | platelet derived growth factor receptor alpha | 4q12 | Cell surface tyrosine kinase receptor for the platelet-derived growth factor family members. | Previously associated with NTD risk in a Hispanic cohort study. | 75 |
| ***PEMT*** | phosphatidylethanolamine N-methyltransferase | 17p11.2 | Converts phosphatidylethanolamine to phosphatidylcholine | Folate one-carbon metabolism. | 1 |
| ***PRICKLE1*** | prickle planar cell polarity protein 1 | 12q12 | Nuclear receptor that may have a role in a negative regulation of the Wnt/beta-catenin signaling pathway. | PCP pathway. NTDs in mouse mutant. | 76 |
| ***PRICKLE2*** | prickle planar cell polarity protein 2 | 3p14.1 | Postsynaptic protein. | PCP pathway. NTDs in mouse mutant. | 77 |
| ***PRICKLE3*** | prickle planar cell polarity protein 3 | Xp11.23 | Protein-binding interface for protein-protein interactions. | PCP pathway. | 78 |
| ***PRICKLE4*** | prickle planar cell polarity protein 4 | 6p21.1 | One of the homologs of Prickle. | PCP pathway. | 79 |
| ***PRKACA*** | protein kinase cAMP-activated catalytic subunit alpha | 19p13.12 | Encodes one of the catalytic subunits of protein kinase A. | NTDs in mouse mutant. | 80 |
| ***PRKACB*** | protein kinase cAMP-activated catalytic subunit beta | 1p31.1 | Encodes a catalytic subunit of cAMP-dependent protein kinase. | NTDs in mouse mutant. | 80 |
| ***PRKCB*** | protein kinase C beta | 16p12.2-p12.1 | Phosphorylate a variety of protein targets. | Role in an inositol prevention of NTD | 81 |
| ***PRKCG*** | protein kinase C gamma | 19q13.42 | Phosphorylate a variety of protein targets. | Role in an inositol prevention of NTD | 81 |
| ***PRMT1*** | protein arginine methyltransferase 1 | 19q13.33 | Methylate arginine residues. | Methylation reactions. | 82 |
| ***PRMT2*** | protein arginine methyltransferase 2 | 21q22.3 | Methylate arginine residues. | Methylation reactions. | 82 |
| ***PTCH1*** | patched 1 | 9q22.32 | Component of the hedgehog signaling pathway. | NTDs in mouse mutant. | 83 |
| ***PTK7*** | protein tyrosine kinase 7 | 6p21.1 | Involved in the Wnt signaling pathway | PCP pathway. NTDs in mouse mutant. Genetic variants reported in human NTD patients. | 84,85 |
| ***RAC1*** | Rac family small GTPase 1 | 7p22.1 | Regulate of cell growth, cytoskeletal reorganization, and the activation of protein kinases. | NTDs in mouse mutant (double heterozygous). | 86 |
| ***RFC1*** | replication factor C subunit 1 | 4p14 | DNA polymerase accessory protein | Previously associated with NTD risk with nominal significance in a Dutch cohort. | 3 |
| ***RNMT*** | RNA guanine-7 methyltransferase | 18p11.21 | Role in 5-prime-terminal capping. | Methylation reactions. | 1 |
| ***ROR2*** | receptor tyrosine kinase like orphan receptor 2 | 9q22.31 | Role in an early formation of the chondrocytes and possibly required for development of the cartilage and growth plate. | PCP pathway. | 87 |
| ***SALL1*** | spalt like transcription factor 1 | 16q12.1 | Zinc finger transcriptional repressor | NTDs in mouse mutant. | 88 |
| ***SALL2*** | spalt like transcription factor 2 | 14q11.2 | Zinc finger transcriptional repressor | NTDs in mouse mutant. | 88 |
| ***SALL4*** | spalt like transcription factor 4 | 20q13.2 | Zinc finger transcriptional repressor | NTDs in mouse mutant. | 88 |
| ***SARDH*** | sarcosine dehydrogenase | 9q34.2 | Catalyses the oxidative demethylation of sarcosine in mitochondrial matrix. | Previously associated with NTD risk with nominal significance in a Dutch cohort. | 3 |
| ***SCRIB*** | scribbled planar cell polarity protein | 8q24.3 | Scaffold protein involved in cell polarization processes | PCP pathway. NTDs in mouse mutant. Genetic variants reported in human NTD patients. | 22,89 |
| ***SDC4*** | syndecan 4 | 20q13.12 | Sulfate proteoglycan | NTDs in mouse mutant (double heterozygous). | 90 |
| ***SEC24B*** | SEC24 homolog B, COPII coat complex component | 4q25 | Vesicle trafficking. | NTDs in mouse mutant. | 91 |
| ***SEMA4C*** | semaphorin 4C | 2q11.2 | Transmembrane and secreted guidance protein. | High-affinity ligand for Plexin-B2 | 92 |
| ***SEPT6*** | septin 6 | Xq24 | Required for cytokinesis. | NTDs in zebrafish mutant. | 93 |

| | | | | | |
|---|---|---|---|---|---|
| ***SESTD1*** | SEC14 and spectrin domain containing 1 | 2q31.2 | Binding partner to Vangl2. | PCP pathway. | 94 |
| ***SETD2*** | SET domain containing 2 | 3p21.31 | Histone methyltransferase | NTDs in mouse mutant. | 95 |
| ***SFRP1*** | secreted frizzled related protein 1 | 8p11.21 | Modulator of Wnt signaling. | NTDs in mouse mutant (double heterozygous). | 96 |
| ***SFRP2*** | secreted frizzled related protein 2 | 4q31.3 | Modulator of Wnt signaling. | NTDs in mouse mutant (double heterozygous). | 96 |
| ***SFRP5*** | secreted frizzled related protein 5 | 10q24.2 | Modulator of Wnt signaling. | PCP pathway. | 97 |
| ***SHH*** | sonic hedgehog | 7q36.3 | Patterning the early embryo. | NTDs in mouse mutant. | 98 |
| ***SHMT1*** | serine hydroxymethyltransferase 1 | 17p11.2 | Reversible conversion of tetrahydrofolate and serine to 5,10-methylene tetrahydrofolate and glycine. | NTDs in mouse mutant. | 99 |
| ***SHROOM1*** | shroom family member 1 | 5q31.1 | Role in the development of the nervous system. | Shroom family protein. | 100 |
| ***SHROOM2*** | shroom family member 2 | Xp22.2 | Role in the development of the nervous system. | Shroom family protein. | 100 |
| ***SHROOM3*** | shroom family member 3 | 4q21.1 | Role in the development of the nervous system. | NTDs in mouse mutant. Genetic variants previously reported in human NTD cases. | 101,102 |
| ***SHROOM4*** | shroom family member 4 | Xp11.22 | Role in the development of the nervous system. | Shroom family protein. | 100 |
| ***SKI*** | SKI proto-oncogene | 1p36.33-p36.32 | Repressor of TGF-beta signaling | NTDs in mouse mutant. | 103 |
| ***SLC2A1*** | solute carrier family 2 member 1 | 1p34.2 | Glucose transporter in the brain, placenta and erythrocytes. | Glucose transport. | 104 |
| ***SLC2A2*** | solute carrier family 2 member 2 | 3q26.2 | Mediates facilitated bidirectional glucose transport. | Glucose transport. NTDs in mouse mutant. | 104 |
| ***SLC40A1*** | solute carrier family 40 member 1 | 2q32.2 | Iron export from epithelial cells. | NTDs in mouse mutant. | 105 |
| ***SMURF1*** | SMAD specific E3 ubiquitin protein ligase 1 | 7q22.1 | Ubiquitin ligase specific for receptor-regulated SMAD proteins in the BMP pathway. | NTDs in mouse mutant. | 106 |
| ***SMURF2*** | SMAD specific E3 ubiquitin protein ligase 2 | 17q23.3-q24.1 | HECT domain E3 ubiquitin ligase. | NTDs in mouse mutant. | 106 |
| ***SNX13*** | sorting nexin 13 | 7p21.1 | Role in intracellular trafficking. | NTDs in mouse mutant. | 107 |
| ***SOD1*** | superoxide dismutase 1 | 21q22.11 | Role in destroying free superoxide radicals. | Oxidative stress. Previously associated with NTD risk in a USA cohort. | 108 |
| ***SOD2*** | superoxide dismutase 2 | 6q25.3 | Role in destroying free superoxide radicals. | Oxidative stress. Previously associated with NTD risk in a USA cohort. | 108 |
| ***SOX3*** | SRY-box 3 | Xq27.1 | Transcription factors involved in the embryonic development regulation. | Previously associated with NTD risk in human cases. | 109 |
| ***SPINT2*** | serine peptidase inhibitor, Kunitz type 2 | 19q13.2 | Inhibits HGF activator which prevents the active hepatocyte growth factor formation. | NTDs in mouse mutant. | 110 |
| ***ST14*** | suppression of tumorigenicity 14 | 11q24.3 | Epithelial-derived, integral membrane serine protease. | NTDs in mouse mutant. | 111 |
| ***T*** | T brachyury transcription factor | 6q27 | Embryonic nuclear transcription factor | Previously associated with NTD risk in human studies. | 1 |
| ***TCN2*** | transcobalamin 2 | 22q12.2 | Binds cobalamin and mediates the cobalamin transport into cells. | Previously associated with NTD risk in an Indian cohort. | 112 |
| ***TFAP2A*** | transcription factor AP-2 alpha | 6p24.3 | Transcription factor | NTDs in mouse mutant. | 113 |
| ***TGIF1*** | TGFB induced factor homeobox 1 | 18p11.31 | Inhibits the retinoic acid receptor binding to a retinoid-responsive promoter. | Retinol metabolism. | 114 |
| ***TMEM231*** | transmembrane protein 231 | 16q23.1 | Role in the formation of the diffusion barrier between the plasma membrane and the cilia. | NTDs in mouse mutant. | 115 |
| ***TMEM67*** | transmembrane protein 67 | 8q22.1 | Functions in formation of the primary cilium. | NTDs in mouse mutant. | 116 |
| ***TP53*** | tumor protein p53 | 17p13.1 | Tumor suppressor gene. | NTDs in mouse mutant. | 117 |
| ***TRDMT1*** | tRNA aspartic acid methyltransferase 1 | 10p13 | Methylation of aspartic acid transfer RNA. | Folate one-carbon metabolism. Previously associated with NTD risk with nominal significance in a Dutch cohort. | 3 |
| ***TULP3*** | tubby like protein 3 | 12p13.33 | Binds to phosphoinositides in the plasma membrane to potentially works as a transcription regulator. | NTDs in mouse mutant. | 118 |
| ***TWIST1*** | twist family bHLH transcription factor 1 | 7p21.1 | Transcription factor with a role in embryonic development. | NTDs in mouse mutant. | 119 |
| ***TXN2*** | thioredoxin 2 | 22q12.3 | Potential roles in the mitochondrial membrane potential regulation and in protection from oxidant-induced apoptosis. | NTDs in mouse mutant. | 120 |
| ***TYMS*** | thymidylate synthetase | 18p11.32 | Catalyses the methylation of deoxyuridylate to deoxythymidylate. | Folate one-carbon metabolism. Pyrimidine synthesis. | 1 |
| ***UCP2*** | uncoupling protein 2 | 11q13.4 | Mitochondrial uncoupling protein with a role in energy metabolism. | Previously associated with NTD risk in a USA cohort. | 121 |
| ***VANGL1*** | VANGL planar cell polarity protein 1 | 1p13.1 | Tetraspanin family member. | PCP pathway. Genetic variants previously reported in human NTD cases. | 122 |
| ***VANGL2*** | VANGL planar cell polarity protein 2 | 1q23.2 | Role in the PCP regulation, especially in the stereociliary bundles of the cochlea. | PCP pathway. NTDs in mouse mutant. | 123 |
| ***WNT11*** | Wnt family member 11 | 11q13.5 | Cell fate regulation and patterning during embryogenesis. | PCP pathway. | 124 |
| ***WNT5A*** | Wnt family member 5A | 3p14.3 | Frizzled-5 and the tyrosine kinase orphan receptor 2 ligand | PCP pathway. NTDs in mouse mutant. | 125 |

| | | | | | |
|---|---|---|---|---|---|
| ***WNT7A*** | Wnt family member 7A | 3p25.1 | Role in the anterior-posterior axis in the female reproductive tract development, and uterine smooth muscle pattering. | PCP pathway. | 126 |
| ***WNT9A*** | Wnt family member 9A | 1q42.13 | Involved in oncogenesis and regulation of cell fate and patterning. | WNT gene family. Role in convergent extension. | 127 |
| ***XRCC1*** | X-ray repair cross complementing 1 | 19q13.31 | Repairs DNA single-strand breaks. | DNA repair gene. | 1 |
| ***XRCC3*** | X-ray repair cross complementing 3 | 14q32.33 | Involved in the homologous recombination for chromosome stability maintenance and DNA damage repair. | DNA repair gene. | 1 |
| ***ZIC1*** | Zic family member 1 | 3q24 | Transcription factor that can transactivate the apolipoprotein E gene. | Brain defects in the mouse mutant. | 128 |
| ***ZIC2*** | Zic family member 2 | 13q32.3 | Transcriptional repressor | NTDs in mouse mutant. | 129 |
| ***ZIC3*** | Zic family member 3 | Xq26.3 | Transcription factor for regulating the left-right body axis formation in the early stage. | NTDs in mouse mutant. | 130,131 |
| ***ZNRF3*** | zinc and ring finger 3 | 22q12.1 | Inhibits Wnt signalling. | NTDs in mouse mutant. | 132 |

**Supplementary Table S2. List of novel/rare genetic variants predicted damaging in NTD cases.**

| ID | Cranio/anen | GT | Position | R | A | Gene | cDNA | Protein | dbSNP | ExAC | Type | Provean | SIFT | PolyPhen | Condel | REVEL | MutationTaster |
|---|---|---|---|---|---|---|---|---|---|---|---|---|---|---|---|---|---|
| 00F176 | Anencephaly | 0/1 | chr3:125828928 | C | T | *ALDH1L1* | c.2236G>A | p.Val746Met | rs145725373 | 6/121254(0.00004948) | missense | Neutral | deleterious | benign | neutral | 0.47 | polymorphism |
| 00F176 | Anencephaly | 0/1 | chr6:150070876 | C | T | *PCMT1* | c.13C>T | p.Arg5Cys | rs553329321 | 2/14004(0.0001428) | missense | Neutral | deleterious | NA | NA | 0.205 | disease causing |
| 00F191 | Anencephaly | 0/1 | chr4:55156627 | CAG | C | *PDGFRA* | c.3029_3030 delAG | p.Arg1011Thrf sTer4 | NA | Novel | frameshift | NA | NA | NA | NA | NA | disease causing |
| 00F191 | Anencephaly | 0/1 | chr9:103002451 | C | T | *INVS* | c.725C>T | p.Ser242Leu | rs2491097 | 553/121356(0.004557) | missense | Deleterious | deleterious | probably damaging | deleterious | 0.597 | disease causing |
| 00F191 | Anencephaly | 0/1 | chr9:136535856 | G | A | *SARDH* | c.2345C>T | p.Ala782Val | rs141409671 | 289/87482(0.003304) | missense | Neutral | tolerated | possibly damaging | deleterious | 0.216 | disease causing |
| 00F378 | Anencephaly | 0/1 | chr11:6653585 | C | G | *DCHS1* | c.3158G>C | p.Trp1053Ser | rs138340204 | 409/121286(0.003372) | missense | Deleterious | deleterious | probably damaging | deleterious | 0.227 | disease causing |
| 00F378 | Anencephaly | 0/1 | chr9:98224170 | C | T | *PTCH1* | c.2671G>A | p.Gly891Ser | rs570091335 | 13/121242(0.0001072) | missense | Deleterious | deleterious | probably damaging | deleterious | 0.849 | disease causing |
| 00F405 | Anencephaly | 0/1 | chr1:228109247 | C | T | *WNT9A* | c.1070G>A | p.Arg357His | rs145836311 | 129/118590(0.001088) | missense | Neutral | deleterious | probably damaging | deleterious | 0.404 | disease causing |
| 00F405 | Anencephaly | 0/1 | chr4:74702951 | C | A | *CXCL6* | c.274C>A | p.Leu92Met | rs140549348 | 1/120966(0.000008267) | missense | Neutral | deleterious | probably damaging | deleterious | 0.189 | disease causing |
| 00F576 | Anencephaly | 0/1 | chr11:71850675 | T | C | *FOLR3* | c.664T>C | p.Ser222Pro | rs2229185 | 17/120682(0.0001409) | missense | Neutral | tolerated | possibly damaging | neutral | 0.301 | polymorphism |
| 00F576 | Anencephaly | 0/1 | chr3:125828823 | T | G | *ALDH1L1* | c.2341A>C | p.Thr781Pro | NA | Novel | missense | Deleterious | deleterious | benign | neutral | 0.24 | disease causing |
| 97F91 | Anencephaly | 0/1 | chr19:45871937 | A | C | *ERCC2* | c.311T>G | p.Phe104Cys | NA | Novel | missense | Deleterious | deleterious | possibly damaging | deleterious | 0.776 | disease causing |
| 97F91 | Anencephaly | 0/1 | chr4:126370186 | A | T | *FAT4* | c.8015A>T | p.Asp2672Val | rs138655269 | 148/120626(0.001227) | missense | Neutral | tolerated | probably damaging | NA | 0.396 | disease causing |
| 01F292 | Anencephaly | 0/1 | chr3:125855674 | A | G | *ALDH1L1* | c.1307T>C | p.Ile436Thr | rs183204130 | 6/83534(0.00007183) | missense | Deleterious | deleterious | probably damaging | deleterious | 0.556 | disease causing |
| 01F292 | Anencephaly | 0/1 | chr3:195966431 | G | A | *PCYT1A* | c.884C>T | p.Pro295Leu | rs774447329 | 2/119768(0.0000167) | missense | Neutral | deleterious | possibly damaging | deleterious | 0.231 | disease causing |
| 01F292 | Anencephaly | 0/1 | chr4:126238305 | C | A | *FAT4* | c.739C>A | p.Pro247Thr | rs191329848 | 465/115352(0.004031) | missense | Deleterious | deleterious | probably damaging | NA | 0.311 | disease causing |
| 01F292 | Anencephaly | 0/1 | chr4:126336725 | C | T | *FAT4* | c.6607C>T | p.Arg2203Trp | rs374328795 | 2/121296(0.00001649) | missense | Deleterious | deleterious | probably damaging | NA | 0.254 | disease causing |
| 01F292 | Anencephaly | 0/1 | chr9:98224206 | C | T | *PTCH1* | c.2635G>A | p.Asp879Asn | NA | 4/121388(0.00003295) | missense | Deleterious | deleterious | probably damaging | deleterious | 0.561 | disease causing |
| 01F292 | Anencephaly | 0/1 | chrX:136648899 | G | T | *ZIC3* | c.49G>T | p.Gly17Cys | rs147232392 | 144/28420(0.005067) | missense | Neutral | deleterious | possibly damaging | deleterious | 0.293 | disease causing |
| 01F29A | CRANIORRHAC HISCHISIS | 0/1 | chr11:6651953 | C | T | *DCHS1* | c.4072G>A | p.Glu1358Lys | rs184586428 | 29/11596(0.002501) | missense | Neutral | deleterious | benign | neutral | 0.168 | polymorphism |
| 01F29A | CRANIORRHAC HISCHISIS | 0/1 | chr7:155596307 | C | T | *SHH* | c.676G>A | p.Ala226Thr | rs104894043 | 3/97762(0.00003069) | missense | Neutral | deleterious | possibly damaging | deleterious | 0.801 | disease causing |
| 01F373 | Anencephaly | 0/1 | chr22:31006935 | G | C | *TCN2* | c.142G>C | p.Glu48Gln | NA | Novel | missense | Neutral | deleterious | benign | deleterious | 0.129 | polymorphism |
| 01F377 | Anencephaly | 0/1 | chr1:156670351 | C | T | *CRABP2* | c.349G>A | p.Asp117Asn | rs146571038 | 60/121386(0.0004943) | missense | Deleterious | deleterious | benign | neutral | 0.165 | disease causing |
| 01F377 | Anencephaly | 0/1 | chr10:17088067 | A | G | *CUBN* | c.3356T>C | p.Leu1119Ser | rs141164907 | 166/121228(0.001369) | missense | Deleterious | tolerated | benign | neutral | 0.178 | polymorphism |
| 01F377 | Anencephaly | 0/1 | chr22:46785380 | C | T | *CELSR1* | c.6362G>A | p.Arg2121His | rs765148329 | 3/118740(0.00002527) | missense | Deleterious | tolerated | benign | neutral | 0.206 | disease causing |
| 01F377 | Anencephaly | 0/1 | chr6:41754013 | C | G | *PRICKLE4* | c.730C>G | p.Arg244Gly | rs141478229 | 238/112016(0.002125) | missense | Deleterious | deleterious | probably damaging | deleterious | 0.623 | disease causing |
| 01F552 | Anencephaly | 0/1 | chr1:109801543 | A | G | *CELSR2* | c.3800A>G | p.His1267Arg | rs138543788 | 874/108766(0.008036) | missense | Deleterious | deleterious | benign | neutral | 0.261 | disease causing |
| 01F552 | Anencephaly | 0/1 | chr14:64892470 | G | A | *MTHFD1* | c.878G>A | p.Arg293His | rs34181110 | 187/121150(0.001544) | missense | Deleterious | deleterious | benign | neutral | 0.242 | disease causing |
| 01F552 | Anencephaly | 0/1 | chr4:77676155 | G | A | *SHROOM3* | c.4519G>A | p.Glu1507Lys | rs141646361 | 205/120414(0.001702) | missense | Neutral | deleterious | possibly damaging | deleterious | 0.231 | disease causing |
| 01F552 | Anencephaly | 0/1 | chr8:104340634 | C | T | *FZD6* | c.1531C>T | p.Arg511Cys | rs151339003 | 1/120596(0.000008292) | missense | Deleterious | deleterious | probably damaging | deleterious | 0.675 | disease causing |
| 01F552 | Anencephaly | 0/1 | chr8:12958149 | G | T | *DLC1* | c.1697C>A | p.Ser566Tyr | rs149628772 | 28/120490(0.0002324) | missense | Neutral | tolerated | possibly damaging | neutral | 0.096 | disease causing |
| 01F571 | Anencephaly | 0/1 | chr10:16955937 | G | A | *CUBN* | c.7406C>T | p.Pro2469Leu | rs202229367 | 8/121128(0.00006605) | missense | Deleterious | tolerated | benign | neutral | 0.248 | disease causing |
| 01F571 | Anencephaly | 0/1 | chr19:44079591 | C | A | *XRCC1* | c.20G>T | p.Arg7Leu | rs2307186 | 206/116620(0.001766) | missense | Deleterious | deleterious | probably damaging | deleterious | 0.261 | disease causing |
| 117F98 | Anencephaly | 0/1 | chr1:109801543 | A | G | *CELSR2* | c.3800A>G | p.His1267Arg | rs138543788 | 874/108766(0.008036) | missense | Deleterious | deleterious | benign | neutral | 0.261 | disease causing |
| 117F98 | Anencephaly | 0/1 | chr11:34485691 | C | T | *CAT* | c.1235C>T | p.Pro412Leu | rs770216105 | 7/121412(0.00005765) | missense | Deleterious | deleterious | probably damaging | deleterious | 0.677 | disease causing |
| 117F98 | Anencephaly | 0/1 | chr11:6648404 | G | A | *DCHS1* | c.5866C>T | p.His1956Tyr | rs755681858 | 2/119776(0.0000167) | missense | Deleterious | deleterious | probably damaging | deleterious | 0.256 | disease causing |
| 117F98 | Anencephaly | 0/1 | chr3:48677191 | G | A | *CELSR3* | c.9827C>T | p.Pro3276Leu | rs61729242 | 901/120304(0.007489) | missense | Deleterious | deleterious | benign | neutral | 0.371 | disease causing |
| 117F98 | Anencephaly | 0/1 | chr7:98649964 | G | C | *SMURF1* | c.585C>G | p.Cys195Trp | rs201268508 | 45/120722(0.0003728) | missense | Neutral | deleterious | benign | deleterious | 0.061 | disease causing |
| 125F05 | Anencephaly | 0/1 | chr1:27106106 | G | A | *ARID1A* | c.5717G>A | p.Arg1906Gln | rs41303631 | 191/121236(0.001575) | missense | Neutral | tolerated | benign | NA | 0.125 | disease causing |
| 125F05 | Anencephaly | 0/1 | chr9:94487152 | C | T | *ROR2* | c.1624G>A | p.Val542Met | rs140213020 | 4/117282(0.00003411) | missense | Neutral | deleterious | possibly damaging | deleterious | 0.451 | disease causing |
| 160F06 | Anencephaly | 0/1 | chr1:109815883 | C | T | *CELSR2* | c.8434C>T | p.Arg2812Trp | rs149683589 | 156/117358(0.001329) | missense | Neutral | deleterious | possibly damaging | deleterious | 0.128 | polymorphism |
| 160F06 | Anencephaly | 0/1 | chr7:17913195 | G | A | *SNX13* | c.709C>T | p.Pro237Ser | rs201830759 | 283/45994(0.006153) | missense | Deleterious | deleterious | probably damaging | deleterious | 0.33 | disease causing |
| 160F06 | Anencephaly | 0/1 | chr8:18080308 | A | T | *NAT1* | c.938A>T | p.Asp313Val | rs56172717 | 292/120548(0.002422) | missense | Deleterious | deleterious | probably damaging | deleterious | 0.255 | disease causing |
| 161F03 | Anencephaly | 0/1 | chr19:44058859 | C | T | *XRCC1* | c.353G>A | p.Arg118Gln | rs762120534 | 2/120292(0.00001663) | missense | Neutral | tolerated | possibly damaging | neutral | 0.121 | disease causing |
| 162F09 | Anencephaly | 0/1 | chr14:21993359 | G | A | *SALL2* | c.503C>T | p.Pro168Leu | rs144885457 | 863/115956(0.007442) | missense | Neutral | deleterious | probably damaging | deleterious | 0.068 | polymorphism |
| 162F09 | Anencephaly | 0/1 | chr21:47570433 | G | A | *FTCD* | c.643C>T | p.Arg215Cys | rs149667449 | 213/120390(0.001769) | missense | Deleterious | deleterious | benign | neutral | 0.385 | disease causing |
| 162F09 | Anencephaly | 0/1 | chr3:125828898 | G | C | *ALDH1L1* | c.2266C>G | p.His756Asp | rs775766256 | 6/121374(0.00004943) | missense | Deleterious | deleterious | possibly damaging | deleterious | 0.774 | disease causing |
| 162F09 | Anencephaly | 0/1 | chr8:144873405 | T | A | *SCRIB* | c.4896A>T | p.Glu1632Asp | rs200643687 | 18/9802(0.001836) | missense | Neutral | deleterious | benign | NA | 0.132 | disease causing |
| 176 | CRANIORRHAC HISCHISIS | 0/1 | chr1:109801265 | C | G | *CELSR2* | c.3522C>G | p.Asp1174Glu | rs369490352 | 1/20726(0.00004825) | missense | Deleterious | tolerated | benign | neutral | 0.279 | disease causing |
| 176 | CRANIORRHAC HISCHISIS | 0/1 | chr11:64575454 | G | A | *MEN1* | c.578C>T | p.Pro193Leu | rs199706698 | 7/121112(0.0000578) | missense | Deleterious | tolerated | benign | neutral | 0.678 | disease causing |
| 176 | CRANIORRHAC HISCHISIS | 0/1 | chr14:59797933 | G | A | *DAAM1* | c.1567G>A | p.Val523Ile | rs750525026 | 7/120928(0.00005789) | missense | Neutral | tolerated | NA | NA | 0.041 | polymorphism |
| 176 | CRANIORRHAC HISCHISIS | 0/1 | chr19:10247822 | A | C | *DNMT1* | c.4428T>G | p.His1476Gln | rs142647321 | 241/92664(0.002601) | missense | Deleterious | tolerated | benign | neutral | 0.247 | polymorphism |
| 176F06 | Anencephaly | 0/1 | chr20:50406762 | C | T | *SALL4* | c.2260G>A | p.Val754Met | rs199607966 | 15/121082(0.0001239) | missense | Neutral | tolerated | possibly damaging | deleterious | 0.045 | polymorphism |
| 176F06 | Anencephaly | 0/1 | chr22:29445447 | C | G | *ZNRF3* | c.1278C>G | p.His426Gln | rs200654061 | 124/118006(0.001051) | missense | Deleterious | deleterious | possibly damaging | deleterious | 0.42 | disease causing |
| 1F07 | Anencephaly | 0/1 | chr1:11854085 | T | A | *MTHFR* | c.1409A>T | p.Glu470Val | rs142617551 | 174/120848(0.00144) | missense | Deleterious | deleterious | benign | neutral | 0.335 | disease causing |
| 1F07 | Anencephaly | 0/1 | chr1:43396804 | G | A | *SLC2A1* | c.188C>T | p.Thr63Met | rs200828053 | 8/121296(0.00006595) | missense | Neutral | tolerated | possibly damaging | neutral | 0.174 | disease causing |
| 1F07 | Anencephaly | 0/1 | chr17:42635177 | C | T | *FZD2* | c.121C>T | p.Pro41Ser | rs776956114 | 1/121036(0.000008262) | missense | Deleterious | tolerated | benign | neutral | 0.697 | disease causing |
| 222F08 | Anencephaly | 0/1 | chr11:6645523 | G | A | *DCHS1* | c.7384C>T | p.Arg2462Trp | rs202186111 | 12/56118(0.0002138) | missense | Neutral | deleterious | probably damaging | deleterious | 0.284 | disease causing |
| 222F08 | Anencephaly | 0/1 | chr14:59835392 | C | T | *DAAM1* | c.3052C>T | p.Arg1018Cys | rs138713289 | 22/112668(0.0001953) | missense | Deleterious | deleterious | probably damaging | deleterious | 0.618 | disease causing |
| 222F08 | Anencephaly | 0/1 | chr3:47127714 | G | A | *SETD2* | c.5368C>T | p.Arg1790Trp | rs774344041 | 1/121288(0.000008245) | missense | Deleterious | deleterious | possibly damaging | deleterious | 0.492 | disease causing |
| 222F98 | Anencephaly | 0/1 | chr11:6661685 | C | T | *DCHS1* | c.1160G>A | p.Arg387His | rs145132459 | 9/121062(0.00007434) | missense | Deleterious | deleterious | probably damaging | deleterious | 0.371 | disease causing |
| 229F08 | Anencephaly | 0/1 | chr10:99531284 | C | T | *SFRP5* | c.307G>A | p.Asp103Asn | rs143647630 | 466/116848(0.003988) | missense | Neutral | tolerated | possibly damaging | neutral | 0.45 | disease causing |
| 229F08 | Anencephaly | 0/1 | chr4:126384816 | C | T | *FAT4* | c.11893C>T | p.Pro3965Ser | NA | Novel | missense | Deleterious | tolerated | benign | NA | 0.642 | disease causing |
| 283F06 | Anencephaly | 0/1 | chr1:11856442 | G | A | *MTHFR* | c.601C>T | p.His201Tyr | NA | Novel | missense | Deleterious | deleterious | probably damaging | deleterious | 0.932 | disease causing |
| 283F06 | Anencephaly | 0/1 | chr9:6554781 | C | A | *GLDC* | c.2203G>T | p.Val735Leu | rs143119940 | 631/91708(0.006881) | missense | Deleterious | deleterious | possibly damaging | deleterious | 0.848 | disease causing |
| 2F07 | Anencephaly | 0/1 | chr22:46760121 | G | A | *CELSR1* | c.8807C>T | p.Pro2936Leu | rs201509338 | 5/117914(0.0000424) | missense | Deleterious | deleterious | probably damaging | deleterious | 0.526 | disease causing |
| 2F07 | Anencephaly | 0/1 | chr3:183887917 | C | T | *DVL3* | c.1622C>T | p.Pro541Leu | NA | 3/250472 (1.228e-5) | missense | Deleterious | tolerated | probably damaging | neutral | 0.173 | disease causing |
| 2F07 | Anencephaly | 0/1 | chr7:51152961 | C | T | *COBL* | c.998G>A | p.Arg333Gln | rs147232176 | 62/120820(0.0005132) | missense | Deleterious | deleterious | probably damaging | deleterious | 0.287 | disease causing |
| 317F06 | Anencephaly | 0/1 | chr22:29442725 | G | A | *ZNRF3* | c.766G>A | p.Val256Met | rs756388076 | 1/117868(0.000008484) | missense | Neutral | tolerated | benign | neutral | 0.14 | disease causing |
| 317F06 | Anencephaly | 0/1 | chr4:126240968 | A | T | *FAT4* | c.3402A>T | p.Glu1134Asp | rs144768563 | 229/120738(0.001897) | missense | Neutral | tolerated | benign | NA | 0.173 | disease causing |
| 335F07 | Anencephaly | 0/1 | chr11:71850766 | T | A | *FOLR3* | c.755T>A | p.Ile252Asn | rs184446689 | 107/121158(0.0008831) | missense | Deleterious | deleterious | probably damaging | deleterious | 0.68 | disease causing |
| 335F07 | Anencephaly | 0/1 | chr4:126329821 | A | G | *FAT4* | c.5792A>G | p.Tyr1931Cys | rs139716832 | 61/121074(0.0005038) | missense | Deleterious | deleterious | probably damaging | NA | 0.518 | disease causing |
| 335F07 | Anencephaly | 0/1 | chr4:126372555 | A | G | *FAT4* | c.10384A>G | p.Ile3462Val | rs147872710 | 261/121260(0.002152) | missense | Neutral | tolerated | benign | NA | 0.183 | disease causing |
| 335F07 | Anencephaly | 0/1 | chr8:104336878 | G | A | *FZD6* | c.544G>A | p.Ala182Thr | rs147788385 | 54/121214(0.0004455) | missense | Neutral | deleterious | probably damaging | deleterious | 0.504 | disease causing |
| 363F04 | Anencephaly | 0/1 | chr12:85674221 | A | G | *ALX1* | c.182A>G | p.His61Arg | rs115440539 | 866/118538(0.007306) | missense | Neutral | deleterious | benign | neutral | 0.253 | disease causing |
| 363F04 | Anencephaly | 0/1 | chr5:7892937 | A | G | *MTRR* | c.1549A>G | p.Thr517Ala | rs41283145 | 563/121410(0.004637) | missense | Deleterious | deleterious | possibly damaging | deleterious | 0.566 | disease causing |
| 363F04 | Anencephaly | 1/1 | chrX:136648899 | G | T | *ZIC3* | c.49G>T | p.Gly17Cys | rs147232392 | 144/28420(0.005067) | missense | Neutral | deleterious | possibly damaging | deleterious | 0.293 | disease causing |
| 384F06 | Anencephaly | 0/1 | chr14:21993363 | G | T | *SALL2* | c.499C>A | p.Pro167Thr | rs150787846 | 102/115978(0.0008795) | missense | Neutral | deleterious | probably damaging | deleterious | 0.15 | polymorphism |
| 389F07 | Anencephaly | 0/1 | chr10:16942755 | A | G | *CUBN* | c.8279T>C | p.Ile2760Thr | rs538864199 | 60/121152(0.0004952) | missense | Deleterious | deleterious | probably damaging | deleterious | 0.438 | disease causing |
| 389F07 | Anencephaly | 0/1 | chr12:3042596 | G | T | *TULP3* | c.709G>T | p.Ala237Ser | rs762869650 | 1 / 121294(0.000008244) | missense | Deleterious | deleterious | possibly damaging | deleterious | 0.854 | disease causing |
| 389F07 | Anencephaly | 0/1 | chr17:54672186 | C | T | *NOG* | c.602C>T | p.Thr201Met | rs766861857 | 4/93592(0.00004274) | missense | Deleterious | deleterious | probably damaging | deleterious | 0.845 | disease causing |
| 389F07 | Anencephaly | 0/1 | chr3:48682550 | C | T | *CELSR3* | c.7890G>A | p.Met2630Ile | rs149614835 | 508/115980(0.00438) | missense | Deleterious | deleterious | probably damaging | deleterious | 0.393 | disease causing |
| 389F07 | Anencephaly | 0/1 | chr5:132160488 | C | T | *SHROOM1* | c.1060G>A | p.Val354Met | rs550046729 | 311/121072(0.002569) | missense | Neutral | tolerated | benign | neutral | 0.015 | polymorphism |
| 392F08 | Anencephaly | 0/1 | chr2:47606179 | T | A | *EPCAM* | c.643T>A | p.Tyr215Asn | rs750826481 | 2/121036(0.00001652) | missense | Deleterious | deleterious | possibly damaging | deleterious | 0.873 | disease causing |
| 392F08 | Anencephaly | 0/1 | chr4:22390109 | C | G | *GPR125* | c.3185G>C | p.Cys1062Ser | rs761749221 | 1/121228(0.000008249) | missense | Deleterious | deleterious | probably damaging | deleterious | 0.56 | disease causing |
| 415F97 | Anencephaly | 0/1 | chr11:6651040 | T | C | *DCHS1* | c.4898A>G | p.Gln1633Arg | rs772857722 | 1/119572(0.000008363) | missense | Deleterious | deleterious | probably damaging | deleterious | 0.226 | disease causing |
| 415F97 | Anencephaly | 0/1 | chr9:6556242 | C | T | *GLDC* | c.2113G>A | p.Val705Met | rs147275962 | 443/121392(0.003649) | missense | Neutral | deleterious | possibly damaging | deleterious | 0.814 | disease causing |
| 415F97 | Anencephaly | 0/1 | chrX:9907369 | C | T | *SHROOM2* | c.4274C>T | p.Ser1425Leu | rs149373874 | 429/64571(0.006644) | missense | Deleterious | deleterious | probably damaging | deleterious | 0.083 | polymorphism |
| 434F98 | Anencephaly | 0/1 | chr13:92345530 | G | C | *GPC5* | c.415G>C | p.Ala139Pro | rs369076704 | 74/121358(0.0006098) | missense | Neutral | tolerated | possibly damaging | neutral | 0.132 | polymorphism |
| 436F98 | Anencephaly | 0/1 | chr1:236208932 | T | C | *NID1* | c.577A>G | p.Ile193Val | NA | Novel | missense | Neutral | tolerated | benign | neutral | 0.217 | disease causing |
| 436F98 | Anencephaly | 0/1 | chr3:125824595 | T | C | *ALDH1L1* | c.2657A>G | p.Lys886Arg | rs142086991 | 47/121408(0.0003871) | missense | Deleterious | deleterious | probably damaging | deleterious | 0.809 | disease causing |
| 439F03 | Anencephaly | 0/1 | chr10:82040481 | G | T | *MAT1A* | c.360C>A | p.Cys120Ter | NA | Novel | stop_gained | NA | NA | NA | NA | NA | disease causing |
| 43F05 | Anencephaly | 0/1 | chr17:26096144 | G | T | *NOS2* | c.1893C>A | p.Tyr631Ter | NA | Novel | stop_gained | NA | NA | NA | NA | NA | disease causing |
| 43F05 | Anencephaly | 0/1 | chr21:34889888 | A | G | *GART* | c.1730T>C | p.Met577Thr | rs151206034 | 123/121234(0.001015) | missense | Deleterious | tolerated | benign | neutral | 0.256 | disease causing |
| 464F03 | Anencephaly | 0/1 | chr17:18244076 | G | A | *SHMT1* | c.571C>T | p.Arg191Cys | rs148701087 | 70/101520(0.0006895) | missense | Deleterious | deleterious | possibly damaging | deleterious | 0.334 | polymorphism |
| 464F03 | Anencephaly | 0/1 | chr5:78417158 | G | A | *BHMT* | c.595G>A | p.Gly199Ser | rs59866108 | 534/121384(0.004399) | missense | Deleterious | tolerated | benign | neutral | 0.288 | disease causing |
| 465F99 | Anencephaly | 0/1 | chr12:85674221 | A | G | *ALX1* | c.182A>G | p.His61Arg | rs115440539 | 866/118538(0.007306) | missense | Neutral | deleterious | benign | neutral | 0.253 | disease causing |
| 465F99 | Anencephaly | 0/1 | chr22:29444445 | C | A | *ZNRF3* | c.981C>A | p.His327Gln | rs55775472 | 589/120608(0.004884) | missense | Deleterious | deleterious | probably damaging | deleterious | 0.704 | disease causing |
| 465F99 | Anencephaly | 0/1 | chr4:126372318 | G | A | *FAT4* | c.10147G>A | p.Gly3383Ser | NA | Novel | missense | Deleterious | deleterious | probably damaging | NA | 0.435 | disease causing |
| 465F99 | Anencephaly | 0/1 | chr7:90894406 | C | T | *FZD1* | c.211C>T | p.Arg71Trp | rs574691354 | 8/986(0.008114) | missense | Neutral | deleterious | possibly damaging | deleterious | 0.361 | disease causing |
| 485F06 | Anencephaly | 0/1 | chr1:1271848 | G | A | *DVL1* | c.1687C>T | p.Arg563Cys | rs202097902 | 34/97602(0.0003484) | missense | Neutral | deleterious | NA | NA | 0.107 | disease causing |

| | | | | | | | | | | | | | | | | | |
|---|---|---|---|---|---|---|---|---|---|---|---|---|---|---|---|---|---|
| 485F06 | Anencephaly | 0/1 | chr11:34477676 | G | C | *CAT* | c.830G>C | p.Trp277Ser | NA | Novel | missense | Deleterious | deleterious | probably damaging | deleterious | 0.908 | disease causing |
| 485F06 | Anencephaly | 0/1 | chr8:41122967 | C | T | *SFRP1* | c.664G>A | p.Asp222Asn | rs140852585 | 3/119790(0.00002504) | missense | Deleterious | deleterious | probably damaging | deleterious | 0.266 | disease causing |
| 486F04 | Anencephaly | 0/1 | chr1:109801543 | A | G | *CELSR2* | c.3800A>G | p.His1267Arg | rs138543788 | 874/108766(0.008036) | missense | Deleterious | deleterious | benign | neutral | 0.261 | disease causing |
| 486F04 | Anencephaly | 0/1 | chr4:126241816 | C | A | *FAT4* | c.4250C>A | p.Pro1417His | rs201515596 | 6/120734(0.0000497) | missense | Deleterious | deleterious | probably damaging | NA | 0.276 | disease causing |
| 486F04 | Anencephaly | 0/1 | chrX:70352035 | G | A | *MED12* | c.4232G>A | p.Ser1411Asn | NA | 1/83499(1.198e-05) | missense | Neutral | tolerated | benign | NA | 0.147 | disease causing |
| 493F99 | Anencephaly | 0/1 | chr1:109795742 | T | A | *CELSR2* | c.3041T>A | p.Leu1014His | rs115856488 | 422/121078(0.003485) | missense | Deleterious | deleterious | probably damaging | deleterious | 0.303 | disease causing |
| 493F99 | Anencephaly | 0/1 | chr17:54671859 | G | A | *NOG* | c.275G>A | p.Gly92Glu | rs199566527 | 176/92530(0.001902) | missense | Neutral | tolerated | probably damaging | neutral | 0.546 | polymorphism |
| 493F99 | Anencephaly | 0/1 | chr6:160113718 | C | G | *SOD2* | c.201G>C | p.Glu67Asp | NA | 1/251836 (4.071e-6) | missense | Deleterious | tolerated | benign | neutral | 0.167 | disease causing |
| 49F04 | Anencephaly | 0/1 | chr14:21991580 | T | C | *SALL2* | c.2282A>G | p.Glu761Gly | rs770681862 | 12/121296(0.00009893) | missense | Neutral | deleterious | possibly damaging | deleterious | 0.044 | disease causing |
| 49F04 | Anencephaly | 0/1 | chr16:75575308 | C | G | *TMEM231* | c.797G>C | p.Arg266Thr | rs199813223 | 129/120206(0.001073) | missense | Deleterious | deleterious | probably damaging | deleterious | 0.403 | disease causing |
| 49F04 | Anencephaly | 0/1 | chr17:41243509 | T | C | *BRCA1* | c.4039A>G | p.Arg1347Gly | rs28897689 | 483/121368(0.00398) | missense | Neutral | tolerated | possibly damaging | deleterious | 0.527 | polymorphism |
| 49F04 | Anencephaly | 0/1 | chr8:12947940 | C | T | *DLC1* | c.3895G>A | p.Glu1299Lys | NA | 1/121142(0.000008255) | missense | Deleterious | deleterious | probably damaging | deleterious | 0.378 | disease causing |
| 500F03 | Anencephaly | 0/1 | chr22:46772993 | C | T | *CELSR1* | c.7549G>A | p.Val2517Met | NA | 1/276358 (3.618e-6) | missense | Neutral | deleterious | probably damaging | deleterious | 0.296 | polymorphism |
| 500F03 | Anencephaly | 0/1 | chr8:18076954 | T | C | *NAT1* | c.136T>C | p.Trp46Arg | NA | 2/19640(0.0001018) | missense | Neutral | deleterious | benign | neutral | 0.041 | polymorphism |
| 508F03 | Anencephaly | 0/1 | chr19:44047814 | G | A | *XRCC1* | c.1738C>T | p.Arg580Trp | rs140655170 | 8/121398(0.0000659) | missense | Deleterious | deleterious | probably damaging | deleterious | 0.199 | polymorphism |
| 508F03 | Anencephaly | 0/1 | chr7:51097255 | G | A | *COBL* | c.1538C>T | p.Ser513Phe | rs61739178 | 981/118914(0.00825) | missense | Deleterious | tolerated | benign | neutral | 0.077 | polymorphism |
| 52F03 | Anencephaly | 0/1 | chr1:1277795 | G | A | *DVL1* | c.310C>T | p.Pro104Ser | rs776308675 | 17/47588(0.0003572) | missense | Deleterious | tolerated | benign | neutral | 0.214 | disease causing |
| 52F03 | Anencephaly | 0/1 | chr1:236180498 | C | T | *NID1* | c.2204G>A | p.Arg735His | rs140374909 | 1055/121408(0.00869) | missense | Deleterious | deleterious | probably damaging | deleterious | 0.575 | disease causing |
| 52F03 | Anencephaly | 0/1 | chr3:47098588 | A | C | *SETD2* | c.6686T>G | p.Val2229Gly | rs377066147 | 306/121230(0.002524) | missense | Neutral | deleterious | benign | neutral | 0.037 | disease causing |
| 52F03 | Anencephaly | 0/1 | chr5:132159914 | G | A | *SHROOM1* | c.1439C>T | p.Pro480Leu | rs151268300 | 214/120928(0.00177) | missense | Deleterious | tolerated | benign | neutral | 0.03 | polymorphism |
| 534F04 | Anencephaly | 0/1 | chr7:150690922 | C | A | *NOS3* | c.31C>A | p.Pro11Thr | rs141170595 | 29/106554(0.0002722) | missense | Neutral | deleterious | possibly damaging | deleterious | 0.13 | polymorphism |
| 534F04 | Anencephaly | 0/1 | chr9:94486491 | G | A | *ROR2* | c.2285C>T | p.Ser762Leu | rs34491822 | 396/121054(0.003271) | missense | Deleterious | deleterious | possibly damaging | deleterious | 0.417 | disease causing |
| 537F03 | Anencephaly | 0/1 | chr1:109794437 | G | C | *CELSR2* | c.1736G>C | p.Gly579Ala | rs200304391 | 22/121142(0.0001816) | missense | Deleterious | tolerated | possibly damaging | deleterious | 0.268 | disease causing |
| 537F03 | Anencephaly | 0/1 | chr12:117768820 | G | A | *NOS1* | c.55C>T | p.Arg19Cys | rs78402290 | 21/120146(0.0001748) | missense | Deleterious | deleterious | probably damaging | deleterious | 0.303 | disease causing |
| 537F03 | Anencephaly | 0/1 | chrX:9914787 | G | A | *SHROOM2* | c.4661G>A | p.Arg1554His | NA | 3/51873(5.783e-05) | missense | Deleterious | deleterious | probably damaging | deleterious | 0.493 | disease causing |
| 556F05 | Anencephaly | 0/1 | chr12:6647018 | C | G | *GAPDH* | c.794C>G | p.Ala265Gly | NA | Novel | missense | Neutral | deleterious | benign | neutral | 0.33 | disease causing |
| 556F05 | Anencephaly | 0/1 | chr9:94486696 | A | G | *ROR2* | c.2080T>C | p.Cys694Arg | rs142386992 | 12/121218(0.000099) | missense | Deleterious | deleterious | probably damaging | deleterious | 0.761 | disease causing |
| 55F08 | Anencephaly | 0/1 | chr17:7577088 | T | A | *TP53* | c.850A>T | p.Thr284Ser | NA | Novel | missense | Neutral | tolerated | possibly damaging | deleterious | 0.495 | polymorphism |
| 55F08 | Anencephaly | 0/1 | chr4:39322991 | G | C | *RFC1* | c.724C>G | p.Arg242Gly | rs760191657 | 1 / 120408(0.000008305) | missense | Deleterious | deleterious | benign | deleterious | 0.081 | disease causing |
| 582F97 | Anencephaly | 0/1 | chr21:48081808 | G | A | *PRMT2* | c.1057G>A | p.Gly353Arg | rs142055128 | 282/65956(0.004276) | missense | Neutral | tolerated | possibly damaging | neutral | 0.04 | disease causing |
| 584F03 | Anencephaly | 0/1 | chr17:54671835 | C | A | *NOG* | c.251C>A | p.Pro84His | rs138481449 | 118/107240(0.0011) | missense | Deleterious | deleterious | probably damaging | deleterious | 0.87 | disease causing |
| 584F03 | Anencephaly | 0/1 | chr22:29446903 | C | A | *ZNRF3* | c.2734C>A | p.Gln912Lys | rs199737518 | 107/100822(0.001061) | missense | Neutral | deleterious | benign | deleterious | 0.14 | disease causing |
| 584F03 | Anencephaly | 0/1 | chr7:51111287 | G | A | *COBL* | c.1199C>T | p.Ser400Leu | NA | 2/121366(0.00001648) | missense | Deleterious | deleterious | probably damaging | deleterious | 0.204 | polymorphism |
| 605F07 | Anencephaly | 0/1 | chr4:126411898 | C | G | *FAT4* | c.13921C>G | p.Gln4641Glu | rs370704263 | 13/121354(0.0001071) | missense | Neutral | tolerated | possibly damaging | NA | 0.228 | disease causing |
| 605F07 | Anencephaly | 0/1 | chr8:12947828 | A | C | *DLC1* | c.4007T>G | p.Val1336Gly | NA | 1/121186(0.000008252) | missense | Neutral | deleterious | possibly damaging | deleterious | 0.387 | disease causing |
| 618F05 | Anencephaly | 0/1 | chr10:16911749 | C | T | *CUBN* | c.9340G>A | p.Gly3114Ser | rs117035284 | 892/121402(0.007347) | missense | Deleterious | deleterious | possibly damaging | deleterious | 0.502 | disease causing |
| 618F05 | Anencephaly | 0/1 | chr22:46762301 | G | A | *CELSR1* | c.8282C>T | p.Ser2761Leu | rs144039991 | 50/19542(0.002559) | missense | Deleterious | deleterious | probably damaging | deleterious | 0.518 | disease causing |
| 618F05 | Anencephaly | 0/1 | chr4:22389381 | C | T | *GPR125* | c.3913G>A | p.Asp1305Asn | NA | 4/245960 (1.626e-5) | missense | Deleterious | deleterious | benign | neutral | 0.236 | disease causing |
| 618F05 | Anencephaly | 0/1 | chr7:17885241 | T | C | *SNX13* | c.1142A>G | p.Asn381Ser | rs555475594 | 9/63206(0.0001424) | missense | Deleterious | tolerated | possibly damaging | deleterious | 0.172 | disease causing |
| 618F05 | Anencephaly | 0/1 | chr7:51095783 | G | C | *COBL* | c.3010C>G | p.Gln1004Glu | rs373944191 | 7/119928(0.00005837) | missense | Neutral | tolerated | benign | neutral | 0.075 | polymorphism |
| 618F05 | Anencephaly | 0/1 | chr8:144875184 | C | T | *SCRIB* | c.3979G>A | p.Val1327Met | rs201563528 | 11/13558(0.0008113) | missense | Neutral | deleterious | probably damaging | NA | 0.07 | disease causing |
| 623F02 | Anencephaly | 0/1 | chr21:44480591 | G | A | *CBS* | c.1105C>T | p.Arg369Cys | rs117687681 | 387/118066(0.003278) | missense | Deleterious | deleterious | probably damaging | deleterious | 0.972 | disease causing |
| 623F02 | Anencephaly | 1/1 | chrX:136648899 | G | T | *ZIC3* | c.49G>T | p.Gly17Cys | rs147232392 | 144/28420(0.005067) | missense | Neutral | deleterious | possibly damaging | deleterious | 0.293 | disease causing |
| 642F05 | Anencephaly | 0/1 | chr11:130059701 | G | A | *ST14* | c.508G>A | p.Glu170Lys | rs150984123 | 220/120788(0.001821) | missense | Neutral | tolerated | possibly damaging | neutral | 0.236 | disease causing |
| 642F05 | Anencephaly | 0/1 | chr14:59113376 | T | C | *DACT1* | c.2035T>C | p.Trp679Arg | rs200977826 | 66/99632(0.0006624) | missense | Deleterious | deleterious | probably damaging | deleterious | 0.296 | disease causing |
| 642F05 | Anencephaly | 0/1 | chr9:6554781 | C | A | *GLDC* | c.2203G>T | p.Val735Leu | rs143119940 | 631/91708(0.006881) | missense | Deleterious | deleterious | possibly damaging | deleterious | 0.848 | disease causing |
| 666F05 | CRANIORRHAC HISCHISIS | 0/1 | chr11:46564053 | C | T | *AMBRA1* | c.1244G>A | p.Arg415His | rs145466300 | 191/121316(0.001574) | missense | Neutral | deleterious | benign | neutral | 0.555 | disease causing |
| 666F05 | CRANIORRHAC HISCHISIS | 0/1 | chr20:32878442 | A | G | *AHCY* | c.769T>C | p.Tyr257His | rs140810436 | 4/121390(0.00003295) | missense | Deleterious | deleterious | probably damaging | deleterious | 0.901 | disease causing |
| 666F05 | CRANIORRHAC HISCHISIS | 0/1 | chr3:64142993 | C | A | *PRICKLE2* | c.445G>T | p.Ala149Ser | rs202025796 | 27/120182(0.0002247) | missense | Deleterious | deleterious | benign | neutral | 0.518 | disease causing |
| 666F05 | CRANIORRHAC HISCHISIS | 0/1 | chr4:74702809 | G | GT | *CXCL6* | c.238_239ins T | p.Val81GlyfsTe r44 | rs564244632 | 689/120548(0.005716) | frameshift | NA | NA | NA | NA | NA | disease causing |
| 666F05 | CRANIORRHAC HISCHISIS | 0/1 | chr9:103002451 | C | T | *INVS* | c.725C>T | p.Ser242Leu | rs2491097 | 553/121356(0.004557) | missense | Deleterious | deleterious | probably damaging | deleterious | 0.597 | disease causing |
| 667F98 | Anencephaly | 0/1 | chr4:126373018 | C | T | *FAT4* | c.10847C>T | p.Thr3616Met | rs111423173 | 473/121236(0.003901) | missense | Deleterious | deleterious | possibly damaging | NA | 0.132 | polymorphism |
| 667F98 | Anencephaly | 0/1 | chr8:12950310 | T | A | *DLC1* | c.3551A>T | p.Gln1184Leu | rs146051142 | 164/115288(0.001423) | missense | Deleterious | deleterious | benign | neutral | 0.206 | disease causing |
| 678F97 | Anencephaly | 0/1 | chr19:7125518 | C | T | *INSR* | c.3034G>A | p.Val1012Met | rs1799816 | 1090/121148(0.008997) | missense | Neutral | deleterious | possibly damaging | deleterious | 0.634 | disease causing |
| 678F97 | Anencephaly | 0/1 | chr7:150707692 | G | C | *NOS3* | c.2693G>C | p.Arg898Pro | NA | 1/36862(0.00002713) | missense | Neutral | tolerated | possibly damaging | deleterious | 0.101 | disease causing |
| 67F06 | Anencephaly | 0/1 | chr4:128626861 | T | C | *INTU* | c.1682T>C | p.Ile561Thr | rs753428033 | 1/121342(0.000008241) | missense | Deleterious | deleterious | possibly damaging | deleterious | 0.588 | disease causing |
| 67F06 | Anencephaly | 0/1 | chr4:74702809 | G | GT | *CXCL6* | c.238_239ins T | p.Val81GlyfsTe r44 | rs564244632 | 689/120548(0.005716) | frameshift | NA | NA | NA | NA | NA | disease causing |
| 67F06 | Anencephaly | 0/1 | chr5:132161279 | C | A | *SHROOM1* | c.554G>T | p.Arg185Leu | NA | 1/670(0.001493) | missense | Deleterious | deleterious | probably damaging | deleterious | 0.26 | disease causing |
| 689F05 | Anencephaly | 0/1 | chr15:80181494 | G | A | *MTHFS* | c.320C>T | p.Thr107Ile | rs140052193 | 107/121332(0.0008819) | missense | Deleterious | deleterious | probably damaging | deleterious | 0.497 | polymorphism |
| 689F05 | Anencephaly | 0/1 | chr2:10095048 | C | T | *GRHL1* | c.25C>T | p.Arg9Trp | NA | 1/252230 (4.064e-6) | missense | Deleterious | deleterious | probably damaging | deleterious | 0.522 | disease causing |
| 693F06 | Anencephaly | 0/1 | chr17:26087106 | C | G | *NOS2* | c.3109G>C | p.Val1037Leu | rs145383683 | 172/116402(0.001478) | missense | Neutral | deleterious | possibly damaging | deleterious | 0.674 | disease causing |
| 693F06 | Anencephaly | 0/1 | chr17:26108102 | C | T | *NOS2* | c.824G>A | p.Gly275Asp | NA | 1/251804 (4.071e-6) | missense | Deleterious | deleterious | probably damaging | deleterious | 0.535 | disease causing |
| 706F07 | Anencephaly | 0/1 | chr1:70881670 | C | T | *CTH* | c.200C>T | p.Thr67Ile | rs28941785 | 786/121194(0.006485) | missense | Deleterious | deleterious | probably damaging | deleterious | 0.931 | disease causing |
| 706F07 | Anencephaly | 0/1 | chr12:85674230 | G | T | *ALX1* | c.191G>T | p.Arg64Leu | rs115596276 | 517/117884(0.004386) | missense | Neutral | tolerated | benign | neutral | 0.509 | disease causing |
| 706F07 | Anencephaly | 0/1 | chr14:64905889 | C | T | *MTHFD1* | c.1673C>T | p.Thr558Met | rs540154396 | 6/117248(0.00005117) | missense | Neutral | tolerated | benign | neutral | 0.044 | disease causing |
| 706F07 | Anencephaly | 0/1 | chr20:50406762 | C | T | *SALL4* | c.2260G>A | p.Val754Met | rs199607966 | 15/121082(0.0001239) | missense | Neutral | tolerated | possibly damaging | deleterious | 0.045 | polymorphism |
| 706F07 | Anencephaly | 0/1 | chr8:104337548 | G | A | *FZD6* | c.1214G>A | p.Arg405Gln | rs150760762 | 229/121404(0.001886) | missense | Neutral | deleterious | probably damaging | deleterious | 0.478 | disease causing |
| 729F98 | Anencephaly | 0/1 | chr2:223160316 | T | C | *PAX3* | c.382A>G | p.Met128Val | rs140921855 | 9/121386(0.00007414) | missense | Neutral | deleterious | benign | neutral | 0.755 | disease causing |
| 729F98 | Anencephaly | 0/1 | chr5:78426800 | G | A | *BHMT* | c.1082G>A | p.Arg361Gln | rs556850126 | 46/121336(0.0003791) | missense | Deleterious | deleterious | probably damaging | deleterious | 0.479 | disease causing |
| 735F97 | Anencephaly | 0/1 | chr1:109801543 | A | G | *CELSR2* | c.3800A>G | p.His1267Arg | rs138543788 | 874/108766(0.008036) | missense | Deleterious | deleterious | benign | neutral | 0.261 | disease causing |
| 735F97 | Anencephaly | 0/1 | chr4:55127448 | G | A | *PDGFRA* | c.236G>A | p.Gly79Asp | rs36035373 | 1062/121400(0.008748) | missense | Deleterious | deleterious | probably damaging | deleterious | 0.308 | disease causing |
| 735F97 | Anencephaly | 0/1 | chr5:132161784 | T | C | *SHROOM1* | c.49A>G | p.Thr17Ala | rs10054043 | 33/29984(0.001101) | missense | Neutral | tolerated | benign | neutral | 0.021 | polymorphism |
| 735F97 | Anencephaly | 0/1 | chr9:6534775 | G | T | *GLDC* | c.2852C>A | p.Ser951Tyr | rs147472391 | 40/117682(0.0003399) | missense | Deleterious | deleterious | possibly damaging | deleterious | 0.645 | disease causing |
| 763F98 | Anencephaly | 0/1 | chr1:228109319 | C | T | *WNT9A* | c.998G>A | p.Arg333Gln | rs745638843 | 1/119718(0.000008353) | missense | Neutral | deleterious | possibly damaging | deleterious | 0.369 | disease causing |
| 763F98 | Anencephaly | 0/1 | chr1:27023366 | C | T | *ARID1A* | c.472C>T | p.Pro158Ser | rs567246585 | 4/1100(0.003636) | missense | Neutral | deleterious | benign | NA | 0.074 | disease causing |
| 84F02 | Anencephaly | 0/1 | chr17:41245027 | G | A | *BRCA1* | c.2521C>T | p.Arg841Trp | rs1800709 | 209/121344(0.001722) | missense | Deleterious | deleterious | benign | neutral | 0.355 | polymorphism |
| 84F02 | Anencephaly | 0/1 | chr7:150707752 | T | G | *NOS3* | c.2753T>G | p.Phe918Cys | NA | 1/70224(0.00001424) | missense | Deleterious | deleterious | probably damaging | deleterious | 0.676 | disease causing |
| 84F02 | Anencephaly | 0/1 | chr8:144887428 | C | T | *SCRIB* | c.2524G>A | p.Gly842Arg | rs571102571 | 1/9440(0.0001059) | missense | Neutral | tolerated | possibly damaging | NA | 0.075 | polymorphism |
| 84F02 | Anencephaly | 0/1 | chrX:136648899 | G | T | *ZIC3* | c.49G>T | p.Gly17Cys | rs147232392 | 144/28420(0.005067) | missense | Neutral | deleterious | possibly damaging | deleterious | 0.293 | disease causing |
| 88F97 | Anencephaly | 0/1 | chr1:236180498 | C | T | *NID1* | c.2204G>A | p.Arg735His | rs140374909 | 1055/121408(0.00869) | missense | Deleterious | deleterious | probably damaging | deleterious | 0.575 | disease causing |
| 88F97 | Anencephaly | 0/1 | chr14:21993363 | G | T | *SALL2* | c.499C>A | p.Pro167Thr | rs150787846 | 102/115978(0.0008795) | missense | Neutral | deleterious | probably damaging | deleterious | 0.15 | polymorphism |
| 88F97 | Anencephaly | 0/1 | chr17:26100230 | G | A | *NOS2* | c.1516C>T | p.Arg506Trp | rs146104261 | 11/121412(0.0000906) | missense | Deleterious | deleterious | possibly damaging | deleterious | 0.108 | polymorphism |
| 88F97 | Anencephaly | 0/1 | chr8:144887545 | C | T | *SCRIB* | c.2407G>A | p.Ala803Thr | NA | Novel | missense | Neutral | deleterious | possibly damaging | NA | 0.118 | disease causing |
| 88F97 | Anencephaly | 0/1 | chr9:103002466 | A | G | *INVS* | c.740A>G | p.Asn247Ser | rs41312220 | 577/121344(0.004755) | missense | Deleterious | tolerated | benign | neutral | 0.108 | disease causing |
| 88F97 | Anencephaly | 1/1 | chrX:9859044 | C | A | *SHROOM2* | c.345C>A | p.His115Gln | NA | Novel | missense | Deleterious | deleterious | possibly damaging | deleterious | 0.171 | disease causing |
| 8F97 | Anencephaly | 0/1 | chr14:59113231 | G | T | *DACT1* | c.1890G>T | p.Lys630Asn | rs201251394 | 18/32030(0.000562) | missense | Neutral | tolerated | possibly damaging | deleterious | 0.086 | polymorphism |
| 8F97 | Anencephaly | 0/1 | chr21:47570433 | G | A | *FTCD* | c.643C>T | p.Arg215Cys | rs149667449 | 213/120390(0.001769) | missense | Deleterious | deleterious | benign | neutral | 0.102 | disease causing |
| 8F97 | Anencephaly | 0/1 | chr7:17915130 | C | A | *SNX13* | c.637G>T | p.Val213Leu | NA | Novel | missense | Neutral | deleterious | benign | deleterious | 0.168 | disease causing |
| 8F97 | Anencephaly | 0/1 | chr8:18080308 | A | T | *NAT1* | c.938A>T | p.Asp313Val | rs56172717 | 292/120548(0.002422) | missense | Deleterious | deleterious | probably damaging | deleterious | 0.255 | disease causing |
| 00F133 | Anencephaly | 0/1 | chr21:34883661 | C | G | *GART* | c.2212G>C | p.Val738Leu | NA | 1/252422 (4.061e-6) | missense | Neutral | deleterious | benign | deleterious | 0.198 | disease causing |
| 00F133 | Anencephaly | 0/1 | chr22:36876729 | T | C | *TXN2* | c.156A>G | p.Ile52Met | rs146269725 | 79/121410(0.0006507) | missense | Neutral | deleterious | benign | neutral | 0.014 | polymorphism |
| 00F133 | Anencephaly | 0/1 | chr4:22394239 | T | G | *GPR125* | c.2556A>C | p.Lys852Asn | rs117922332 | 1209/121312(0.009966) | missense | Deleterious | deleterious | probably damaging | deleterious | 0.292 | disease causing |
| 99F553 | Anencephaly | 0/1 | chr10:16949566 | G | A | *CUBN* | c.7646C>T | p.Thr2549Met | rs537292240 | 11/121390(0.00009062) | missense | Deleterious | deleterious | probably damaging | deleterious | 0.296 | disease causing |
| 99F553 | Anencephaly | 0/1 | chr15:58285237 | A | G | *ALDH1A2* | c.590T>C | p.Ile197Thr | NA | Novel | missense | Deleterious | deleterious | probably damaging | deleterious | 0.384 | disease causing |
| 99F553 | Anencephaly | 0/1 | chr5:78417158 | G | A | *BHMT* | c.595G>A | p.Gly199Ser | rs59866108 | 534/121384(0.004399) | missense | Deleterious | tolerated | benign | neutral | 0.288 | disease causing |
| 99F553 | Anencephaly | 0/1 | chr7:51095669 | G | A | *COBL* | c.3124C>T | p.Arg1042Cys | rs370348190 | 7/117254(0.0000597) | missense | Neutral | tolerated | benign | neutral | 0.028 | polymorphism |
| 99F590 | Anencephaly | 0/1 | chr4:55127448 | G | A | *PDGFRA* | c.236G>A | p.Gly79Asp | rs36035373 | 1062/121400(0.008748) | missense | Deleterious | deleterious | probably damaging | deleterious | 0.308 | disease causing |
| 99F643 | Anencephaly | 0/1 | chr6:99824049 | G | A | *COQ3* | c.496C>T | p.Arg166Trp | rs375375802 | 5/121006(0.00004132) | missense | Deleterious | deleterious | probably damaging | deleterious | 0.512 | disease causing |
| 99F643 | Anencephaly | 0/1 | chr9:136573437 | C | T | *SARDH* | c.1442G>A | p.Arg481His | rs35699831 | 84/120618(0.0006964) | missense | Deleterious | deleterious | probably damaging | deleterious | 0.831 | disease causing |
| f10-505 | Anencephaly | 0/1 | chr1:183095324 | G | T | *LAMC1* | c.2871G>T | p.Gln957His | rs201601443 | 133/121336(0.001096) | missense | Neutral | tolerated | benign | neutral | 0.056 | disease causing |
| f10-505 | Anencephaly | 0/1 | chr8:102571003 | G | A | *GRHL2* | c.641G>A | p.Ser214Asn | rs199931364 | 8/118074(0.00006775) | missense | Neutral | deleterious | probably damaging | deleterious | 0.217 | NA |
| f11-278 | Anencephaly | 0/1 | chr10:16943450 | C | T | *CUBN* | c.8071G>A | p.Gly2691Arg | rs1801237 | 32/121228(0.000264) | missense | Deleterious | deleterious | benign | neutral | 0.525 | disease causing |

| | | | | | | | | | | | | | | | | | |
|---|---|---|---|---|---|---|---|---|---|---|---|---|---|---|---|---|---|
| f11-278 | Anencephaly | 0/1 | chr12:85674229 | C | T | *ALX1* | c.190C>T | p.Arg64Cys | rs145944049 | 208/118060(0.001762) | missense | Neutral | tolerated | benign | neutral | 0.534 | disease causing |
| f11-278 | Anencephaly | 0/1 | chr3:48680471 | G | C | *CELSR3* | c.8335C>G | p.Arg2779Gly | NA | Novel | missense | Deleterious | deleterious | benign | neutral | 0.306 | disease causing |
| f11-278 | Anencephaly | 0/1 | chr3:48690482 | G | A | *CELSR3* | c.5587C>T | p.Arg1863Trp | rs142348413 | 11/120056(0.00009162) | missense | Neutral | deleterious | possibly damaging | deleterious | 0.379 | disease causing |
| f11-278 | Anencephaly | 0/1 | chr4:77659945 | C | T | *SHROOM3* | c.619C>T | p.His207Tyr | rs139272770 | 436/121202(0.003597) | missense | Deleterious | deleterious | possibly damaging | deleterious | 0.257 | disease causing |
| f11-278 | Anencephaly | 0/1 | chr6:32946110 | G | A | *BRD2* | c.1786G>A | p.Gly596Ser | NA | 2/115658(0.00001729) | missense | Neutral | tolerated | possibly damaging | neutral | 0.043 | polymorphism |
| f92-322 | Anencephaly | 0/1 | chr10:17032415 | G | A | *CUBN* | c.4268C>T | p.Thr1423Met | rs483352704 | 1/121410(0.000008237) | missense | Neutral | deleterious | probably damaging | deleterious | 0.331 | polymorphism |
| f93-3 | Anencephaly | 0/1 | chr20:32883389 | C | T | *AHCY* | c.31G>A | p.Asp11Asn | rs776007050 | 4/110216(0.00003629) | missense | Deleterious | deleterious | benign | deleterious | 0.62 | disease causing |
| f93-3 | Anencephaly | 0/1 | chr8:144890990 | T | C | *SCRIB* | c.1904A>G | p.Asp635Gly | NA | Novel | missense | Deleterious | deleterious | benign | NA | 0.041 | polymorphism |
| f93-80 | Anencephaly | 0/1 | chr1:109794593 | C | T | *CELSR2* | c.1892C>T | p.Thr631Met | rs41279706 | 474/121308(0.003907) | missense | Deleterious | deleterious | probably damaging | deleterious | 0.249 | disease causing |
| f93-80 | Anencephaly | 0/1 | chr6:43098128 | A | G | *PTK7* | c.655A>G | p.Ser219Gly | NA | Novel | missense | Neutral | tolerated | benign | neutral | 0.418 | disease causing |
| f93-9 | Anencephaly | 0/1 | chr1:1273712 | G | A | *DVL1* | c.1369C>T | p.His457Tyr | NA | 1/251670 (4.073e-6) | missense | Deleterious | deleterious | possibly damaging | deleterious | 0.565 | polymorphism |
| f93-9 | Anencephaly | 0/1 | chr14:21993363 | G | T | *SALL2* | c.499C>A | p.Pro167Thr | rs150787846 | 102/115978(0.0008795) | missense | Neutral | deleterious | probably damaging | deleterious | 0.15 | polymorphism |
| f94-114 | Anencephaly | 0/1 | chr1:27106106 | G | A | *ARID1A* | c.5717G>A | p.Arg1906Gln | rs41303631 | 191/121236(0.001575) | missense | Neutral | tolerated | benign | NA | 0.125 | disease causing |
| f94-114 | Anencephaly | 0/1 | chr14:37050297 | T | C | *NKX2-8* | c.530A>G | p.Asp177Gly | NA | Novel | missense | Deleterious | deleterious | possibly damaging | deleterious | 0.652 | disease causing |
| f94-114 | Anencephaly | 0/1 | chr3:195969442 | T | C | *PCYT1A* | c.556A>G | p.Lys186Glu | NA | 3/252220 (1.219e-5) | missense | Deleterious | deleterious | probably damaging | deleterious | 0.939 | disease causing |
| f94-114 | Anencephaly | 0/1 | chr3:47162995 | C | T | *SETD2* | c.3131G>A | p.Ser1044Asn | rs587778673 | 1/121274(0.000008246) | missense | Neutral | deleterious | benign | deleterious | 0.212 | disease causing |
| f94-258 | CRANIORRHAC HISCHISIS | 0/1 | chr4:126389831 | C | T | *FAT4* | c.12064C>T | p.Arg4022Trp | rs138019311 | 516/121342(0.004252) | missense | Deleterious | deleterious | benign | NA | 0.489 | disease causing |
| f95-60 | CRANIORRHAC HISCHISIS | 0/1 | chr1:109793077 | C | A | *CELSR2* | c.376C>A | p.Gln126Lys | rs62623708 | 352/120490(0.002921) | missense | Neutral | deleterious | benign | neutral | 0.102 | polymorphism |
| f95-60 | CRANIORRHAC HISCHISIS | 0/1 | chr9:6605157 | G | T | *GLDC* | c.835C>A | p.Leu279Ile | NA | 3/282646 (1.082e-5) | missense | Neutral | tolerated | possibly damaging | neutral | 0.529 | disease causing |
| f95-60 | CRANIORRHAC HISCHISIS | 1/1 | chrX:50438789 | C | T | *SHROOM4* | c.266G>A | p.Arg89Lys | rs148911180 | 180/87455(0.002058) | missense | Neutral | deleterious | possibly damaging | deleterious | 0.254 | polymorphism |
| f95-83 | Anencephaly | 0/1 | chr4:126241503 | C | T | *FAT4* | c.3937C>T | p.Pro1313Ser | rs748743944 | 3/120732(0.00002485) | missense | Deleterious | deleterious | probably damaging | NA | 0.69 | disease causing |
| f95-83 | Anencephaly | 0/1 | chr6:15496930 | C | T | *JARID2* | c.1474C>T | p.Arg492Cys | rs150448457 | 1021/117660(0.008678) | missense | Neutral | deleterious | possibly damaging | deleterious | 0.424 | disease causing |
| f96-104 | Anencephaly | 0/1 | chr1:109801543 | A | G | *CELSR2* | c.3800A>G | p.His1267Arg | rs138543788 | 874/108766(0.008036) | missense | Deleterious | deleterious | benign | neutral | 0.261 | disease causing |
| f96-104 | Anencephaly | 0/1 | chr9:103002451 | C | T | *INVS* | c.725C>T | p.Ser242Leu | rs2491097 | 553/121356(0.004557) | missense | Deleterious | deleterious | probably damaging | deleterious | 0.597 | disease causing |
| f96-341 | Anencephaly | 0/1 | chr9:94486491 | G | A | *ROR2* | c.2285C>T | p.Ser762Leu | rs34491822 | 396/121054(0.003271) | missense | Deleterious | deleterious | possibly damaging | deleterious | 0.417 | disease causing |
| f96-425 | Anencephaly | 0/1 | chr10:17204222 | C | T | *TRDMT1* | c.266G>A | p.Gly89Asp | rs138942626 | 74/121328(0.0006099) | missense | Neutral | deleterious | probably damaging | deleterious | 0.25 | disease causing |
| f96-425 | Anencephaly | 0/1 | chr3:48677191 | G | A | *CELSR3* | c.9827C>T | p.Pro3276Leu | rs61729242 | 901/120304(0.007489) | missense | Deleterious | deleterious | benign | neutral | 0.128 | disease causing |
| f96-425 | Anencephaly | 0/1 | chr6:15496930 | C | T | *JARID2* | c.1474C>T | p.Arg492Cys | rs150448457 | 1021/117660(0.008678) | missense | Neutral | deleterious | possibly damaging | deleterious | 0.424 | disease causing |
| f96-59 | Anencephaly | 0/1 | chr18:13752358 | T | G | *RNMT* | c.1291T>G | p.Ser431Ala | rs149656559 | 9/120960(0.0000744) | missense | Neutral | tolerated | probably damaging | deleterious | 0.279 | disease causing |
| f96-59 | Anencephaly | 0/1 | chr22:46772993 | C | T | *CELSR1* | c.7549G>A | p.Val2517Met | NA | 1/276358 (3.618e-6) | missense | Neutral | deleterious | probably damaging | deleterious | 0.296 | polymorphism |
| f96-59 | Anencephaly | 0/1 | chr6:151281448 | C | T | *MTHFD1L* | c.1841C>T | p.Ala614Val | rs202038463 | 57/120504(0.000473) | missense | Deleterious | deleterious | possibly damaging | deleterious | 0.554 | disease causing |

: Samples carrying more than one rare damaging variants.

: Genes carrying more than one rare damaging variants.

**Supplementary Table S3. List of novel/rare genetic variants predicted damaging in the NTD capture controls.**

| ID | Condition | GT | Position | R | A | Gene | cDNA | Protein | dbSNP | ExAC | Type | Provean | SIFT | PolyPhen | Condel | REVEL | MutationTas |
|---|---|---|---|---|---|---|---|---|---|---|---|---|---|---|---|---|---|
| 11Mother | Healthy | 0/1 | 1:109815529 | T | [A] | *CELSR2* | c.8218T>A | p.Ser2740Thr | NA | Novel | missense | neutral | deleterious | probably damaging | deleterious | 0.19 | disease causing |
| 11Mother | Healthy | 0/1 | 3:170732426 | G | [A] | *SLC2A2* | c.203C>T | p.Pro68Leu | rs7637863 | 0.004234 (514 / 121390) | missense | neutral | deleterious | benign | neutral | 0.289 | disease causing |
| 11Mother | Healthy | 0/1 | 6:41751219 | T | [G] | *PRICKLE4* | c.8T>G | p.Val3Gly | rs150368829 | 0.003033 (352 / 116038) | missense | deleterious | deleterious | benign | neutral | 0.123 | polymorphism |
| 11Mother | Healthy | 0/1 | 6:43044640 | G | [A] | *PTK7* | c.19G>A | p.Gly7Arg | rs139636574 | 0.003618 (37 / 10226) | missense | neutral | deleterious | benign | neutral | 0.132 | polymorphism |
| 11Father | Healthy | 0/1 | 5:78426913 | GA | [G] | *BHMT* | c.1196delA | p.Lys400Asnfs | rs763726268 | 0.0002288 | frameshift | NA | NA | NA | NA | NA | NA |
| 8Father | Healthy | 0/1 | 8:12957657 | C | [T] | *DLC1* | c.2189G>A | p.Arg730Gln | rs140340878 | 0.001211 (147 / 121360) | missense | neutral | tolerated | probably damaging | deleterious | 0.095 | disease causing |
| 8Mother | Healthy | 0/1 | 19:44056386 | C | [G] | *XRCC1* | c.865G>C | p.Ala289Pro | rs745463797 | 0.0001131 (31/274188) | missense | neutral | tolerated | possibly damaging | neutral | 0.088 | disease causing |

**Supplementary Table S4. Sanger sequencing confirmation of the *FAT4* and *COBL* rare variants.**

| ID | Cranio/anen | GT | Position | R | A | Gene | Electropherograms | cDNA | Protein | dbSNP | ExAC | Type |
|---|---|---|---|---|---|---|---|---|---|---|---|---|
| **97F91** | Anencephaly | 0/1 | chr4:126370186 | A | T | *FAT4* | 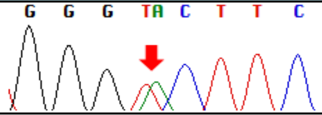  | c.8015A>T | p.Asp2672Val | rs138655269 | 148/120626(0.001227) | missense |
| **01F292** | Anencephaly | 0/1 | chr4:126238305 | C | A | *FAT4* | 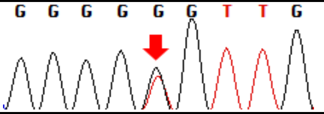  | c.739C>A | p.Pro247Thr | rs191329848 | 465/115352(0.004031) | missense |
| **01F292** | Anencephaly | 0/1 | chr4:126336725 | C | T | *FAT4* | 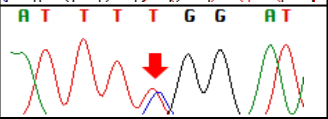  | c.6607C>T | p.Arg2203Trp | rs374328795 | 2/121296(0.00001649) | missense |
| **229F08** | Anencephaly | 0/1 | chr4:126384816 | C | T | *FAT4* | 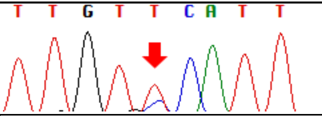  | c.11893C>T | p.Pro3965Ser | NA | Novel | missense |
| **317F06** | Anencephaly | 0/1 | chr4:126240968 | A | T | *FAT4* | 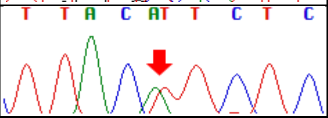  | c.3402A>T | p.Glu1134Asp | rs144768563 | 229/120738(0.001897) | missense |
| **335F07** | Anencephaly | 0/1 | chr4:126329821 | A | G | *FAT4* | 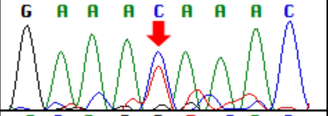  | c.5792A>G | p.Tyr1931Cys | rs139716832 | 61/121074(0.0005038) | missense |
| **335F07** | Anencephaly | 0/1 | chr4:126372555 | A | G | *FAT4* | 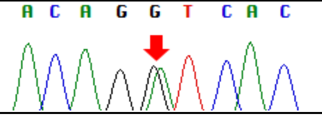  | c.10384A>G | p.Ile3462Val | rs147872710 | 261/121260(0.002152) | missense |
| **465F99** | Anencephaly | 0/1 | chr4:126372318 | G | A | *FAT4* | 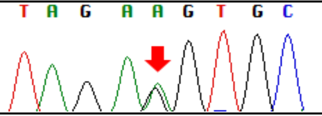  | c.10147G>A | p.Gly3383Ser | NA | Novel | missense |
| **486F04** | Anencephaly | 0/1 | chr4:126241816 | C | A | *FAT4* | 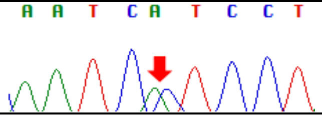  | c.4250C>A | p.Pro1417His | rs201515596 | 6/120734(0.0000497) | missense |
| **605F07** | Anencephaly | 0/1 | chr4:126411898 | C | G | *FAT4* | 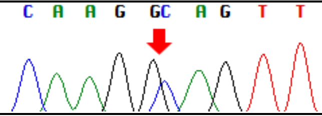  | c.13921C>G | p.Gln4641Glu | rs370704263 | 13/121354(0.0001071) | missense |
| **667F98** | Anencephaly | 0/1 | chr4:126373018 | C | T | *FAT4* | 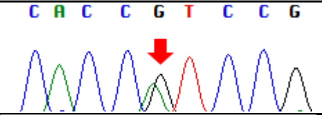  | c.10847C>T | p.Thr3616Met | rs111423173 | 473/121236(0.003901) | missense |
| **f94-258** | CRANIORRHACHISCHISIS | 0/1 | chr4:126389831 | C | T | *FAT4* | 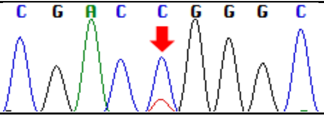  | c.12064C>T | p.Arg4022Trp | rs138019311 | 516/121342(0.004252) | missense |
| **f95-83** | Anencephaly | 0/1 | chr4:126241503 | C | T | *FAT4* | 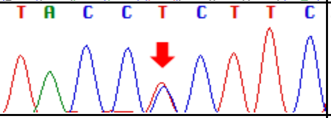  | c.3937C>T | p.Pro1313Ser | rs748743944 | 3/120732(0.00002485) | missense |
| **2F07** | Anencephaly | 0/1 | chr7:51152961 | C | T | *COBL* | 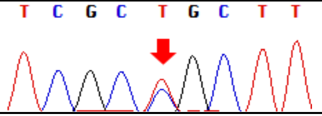  | c.998G>A | p.Arg333Gln | rs147232176 | 62/120820(0.0005132) | missense |
| **508F03** | Anencephaly | 0/1 | chr7:51097255 | G | A | *COBL* | 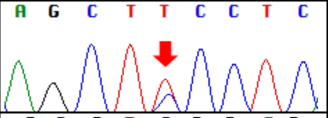  | c.1538C>T | p.Ser513Phe | rs61739178 | 981/118914(0.00825) | missense |
| **584F03** | Anencephaly | 0/1 | chr7:51111287 | G | A | *COBL* | 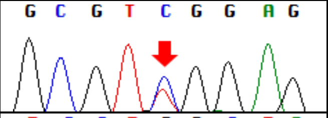  | c.1199C>T | p.Ser400Leu | NA | 2/121366(0.00001648) | missense |
| **618F05** | Anencephaly | 0/1 | chr7:51095783 | G | C | *COBL* | 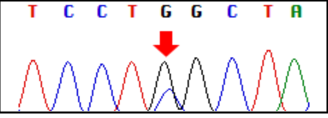  | c.3010C>G | p.Gln1004Glu | rs373944191 | 7/119928(0.00005837) | missense |

| | | | | | | | | | | | | |
|---|---|---|---|---|---|---|---|---|---|---|---|---|
| **99F553** | Anencephaly | 0/1 | chr7:51095669 | G | A | *COBL* | 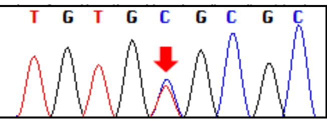  | c.3124C>T | p.Arg1042Cys | rs370348190 | 7/117254(0.0 000597) | missense |

Genetic variants confirmed by Sanger sequence are indicated by red arrows.

**REFERENCES (Supplementary Tables)**


1. Greene ND, Stanier P, Copp AJ. Genetics of human neural tube defects. Hum Mol Genet 2009: 18: R113-129.
2. Deak KL, Dickerson ME, Linney E et al. Analysis of ALDH1A2, CYP26A1, CYP26B1, CRABP1, and CRABP2 in human neural tube defects suggests a possible association with alleles in ALDH1A2. Birth Defects Res A Clin Mol Teratol 2005: 73: 868-875.
3. Franke B, Vermeulen SH, Steegers-Theunissen RP et al. An association study of 45 folate-related genes in spina bifida: Involvement of cubilin (CUBN) and tRNA aspartic acid methyltransferase 1 (TRDMT1). Birth Defects Res A Clin Mol Teratol 2009: 85: 216-226.
4. Wu L, Lu X, Guo J et al. Association between ALDH1L1 gene polymorphism and neural tube defects in the Chinese Han population. Neurol Sci 2016: 37: 1049-1054.
5. Zhao Q, Behringer RR, de Crombrugghe B. Prenatal folic acid treatment suppresses acrania and meroanencephaly in mice mutant for the Cart1 homeobox gene. Nat Genet 1996: 13: 275-283.
6. Fimia GM, Stoykova A, Romagnoli A et al. Ambra1 regulates autophagy and development of the nervous system. Nature 2007: 447: 1121-1125.
7. Narisawa A, Komatsuzaki S, Kikuchi A et al. Mutations in genes encoding the glycine cleavage system predispose to neural tube defects in mice and humans. Hum Mol Genet 2012: 21: 1496-1503.
8. Olshan AF, Shaw GM, Millikan RC et al. Polymorphisms in DNA repair genes as risk factors for spina bifida and orofacial clefts. Am J Med Genet A 2005: 135: 268-273.
9. Chandler RL, Brennan J, Schisler JC et al. ARID1a-DNA interactions are required for promoter occupancy by SWI/SNF. Mol Cell Biol 2013: 33: 265-280.
10. Eichers ER, Abd-El-Barr MM, Paylor R et al. Phenotypic characterization of Bbs4 null mice reveals age-dependent penetrance and variable expressivity. Hum Genet 2006: 120: 211-226.
11. Zhu H, Curry S, Wen S et al. Are the betaine-homocysteine methyltransferase (BHMT and BHMT2) genes risk factors for spina bifida and orofacial clefts? Am J Med Genet A 2005: 135: 274-277.
12. Boyles AL, Billups AV, Deak KL et al. Neural tube defects and folate pathway genes: family-based association tests of gene-gene and gene-environment interactions. Environ Health Perspect 2006: 114: 1547-1552.
13. Cao L, Wang Y, Zhang R et al. Association of neural tube defects with gene polymorphisms in one-carbon metabolic pathway. Childs Nerv Syst 2017.
14. Zwerts F, Lupu F, De Vriese A et al. Lack of endothelial cell survivin causes embryonic defects in angiogenesis, cardiogenesis, and neural tube closure. Blood 2007: 109: 4742-4752.
15. Castranio T, Mishina Y. Bmp2 is required for cephalic neural tube closure in the mouse. Dev Dyn 2009: 238: 110-122.
16. Winnier G, Blessing M, Labosky PA et al. Bone morphogenetic protein-4 is required for mesoderm formation and patterning in the mouse. Genes Dev 1995: 9: 2105-2116.
17. Wang X, Wang RH, Li W et al. Genetic interactions between Brca1 and Gadd45a in centrosome duplication, genetic stability, and neural tube closure. J Biol Chem 2004: 279: 29606-29614.
18. Gyuris A, Donovan DJ, Seymour KA et al. The chromatin-targeting protein Brd2 is required for neural tube closure and embryogenesis. Biochim Biophys Acta 2009: 1789: 413-421.

19. Arslan M, Melek M, Demir H et al. Relationship of antioxidant enzyme activities with myelomeningocele. Turk Neurosurg 2012: 22: 300-304.
20. Allache R, De Marco P, Merello E et al. Role of the planar cell polarity gene CELSR1 in neural tube defects and caudal agenesis. Birth Defects Res A Clin Mol Teratol 2012: 94: 176-181.
21. Curtin JA, Quint E, Tsipouri V et al. Mutation of Celsr1 disrupts planar polarity of inner ear hair cells and causes severe neural tube defects in the mouse. Curr Biol 2003: 13: 1129-1133.
22. Robinson A, Escuin S, Doudney K et al. Mutations in the planar cell polarity genes CELSR1 and SCRIB are associated with the severe neural tube defect craniorachischisis. Hum Mutat 2012: 33: 440-447.
23. Tissir F, Goffinet AM. Shaping the nervous system: role of the core planar cell polarity genes. Nat Rev Neurosci 2013: 14: 525-535.
24. Grego-Bessa J, Hildebrand J, Anderson KV. Morphogenesis of the mouse neural plate depends on distinct roles of cofilin 1 in apical and basal epithelial domains. Development 2015: 142: 1305-1314.
25. Zhu H, Enaw JO, Ma C et al. Association between CFL1 gene polymorphisms and spina bifida risk in a California population. BMC Med Genet 2007: 8: 12.
26. Enaw JO, Zhu H, Yang W et al. CHKA and PCYT1A gene polymorphisms, choline intake and spina bifida risk in a California population. BMC Med 2006: 4: 36.
27. Barbera JP, Rodriguez TA, Greene ND et al. Folic acid prevents exencephaly in Cited2 deficient mice. Hum Mol Genet 2002: 11: 283-293.
28. Carroll EA, Gerrelli D, Gasca S et al. Cordon-bleu is a conserved gene involved in neural tube formation. Dev Biol 2003: 262: 16-31.
29. Yamamoto S, Nishimura O, Misaki K et al. Cthrc1 selectively activates the planar cell polarity pathway of Wnt signaling by stabilizing the Wnt-receptor complex. Dev Cell 2008: 15: 23-36.
30. Shi M, Caprau D, Romitti P et al. Genotype frequencies and linkage disequilibrium in the CEPH human diversity panel for variants in folate pathway genes MTHFR, MTHFD, MTRR, RFC1, and GCP2. Birth Defects Res A Clin Mol Teratol 2003: 67: 545-549.
31. Habas R, Kato Y, He X. Wnt/Frizzled activation of Rho regulates vertebrate gastrulation and requires a novel Formin homology protein Daam1. Cell 2001: 107: 843-854.
32. Suriben R, Kivimae S, Fisher DA et al. Posterior malformations in Dact1 mutant mice arise through misregulated Vangl2 at the primitive streak. Nat Genet 2009: 41: 977-985.
33. Shi Y, Ding Y, Lei YP et al. Identification of novel rare mutations of DACT1 in human neural tube defects. Hum Mutat 2012: 33: 1450-1455.
34. Mao Y, Mulvaney J, Zakaria S et al. Characterization of a Dchs1 mutant mouse reveals requirements for Dchs1-Fat4 signaling during mammalian development. Development 2011: 138: 947-957.
35. Sabbir MG, Wigle N, Loewen S et al. Identification and characterization of Dlc1 isoforms in the mouse and study of the biological function of a single gene trapped isoform. BMC Biol 2010: 8: 17.
36. Bilder D. Epithelial polarity and proliferation control: links from the Drosophila neoplastic tumor suppressors. Genes Dev 2004: 18: 1909-1925.
37. Takebayashi S, Tamura T, Matsuoka C et al. Major and essential role for the DNA methylation mark in mouse embryogenesis and stable association of DNMT1 with newly replicated regions. Mol Cell Biol 2007: 27: 8243-8258.
38. Hamblet NS, Lijam N, Ruiz-Lozano P et al. Dishevelled 2 is essential for cardiac outflow tract development, somite segmentation and neural tube closure. Development 2002: 129: 5827-5838.

39. Pyrgaki C, Liu A, Niswander L. Grainyhead-like 2 regulates neural tube closure and adhesion molecule expression during neural fold fusion. Dev Biol 2011: 353: 38-49.
40. Coin F, Marinoni JC, Rodolfo C et al. Mutations in the XPD helicase gene result in XP and TTD phenotypes, preventing interaction between XPD and the p44 subunit of TFIIH. Nat Genet 1998: 20: 184-188.
41. Saburi S, Hester I, Fischer E et al. Loss of Fat4 disrupts PCP signaling and oriented cell division and leads to cystic kidney disease. Nat Genet 2008: 40: 1010-1015.
42. Rock R, Schrauth S, Gessler M. Expression of mouse dchs1, fjx1, and fat-j suggests conservation of the planar cell polarity pathway identified in Drosophila. Dev Dyn 2005: 234: 747-755.
43. Piedrahita JA, Oetama B, Bennett GD et al. Mice lacking the folic acid-binding protein Folbp1 are defective in early embryonic development. Nat Genet 1999: 23: 228-232.
44. O'Byrne MR, Au KS, Morrison AC et al. Association of folate receptor (FOLR1, FOLR2, FOLR3) and reduced folate carrier (SLC19A1) genes with meningomyelocele. Birth Defects Res A Clin Mol Teratol 2010: 88: 689-694.
45. Spiegelstein O, Eudy JD, Finnell RH. Identification of two putative novel folate receptor genes in humans and mouse. Gene 2000: 258: 117-125.
46. Lowe KE, Osborne CB, Lin BF et al. Regulation of folate and one-carbon metabolism in mammalian cells. II. Effect of folylpoly-gamma-glutamate synthetase substrate specificity and level on folate metabolism and folylpoly-gamma-glutamate specificity of metabolic cycles of one-carbon metabolism. J Biol Chem 1993: 268: 21665-21673.
47. Gray RS, Abitua PB, Wlodarczyk BJ et al. The planar cell polarity effector Fuz is essential for targeted membrane trafficking, ciliogenesis and mouse embryonic development. Nat Cell Biol 2009: 11: 1225-1232.
48. Yu H, Smallwood PM, Wang Y et al. Frizzled 1 and frizzled 2 genes function in palate, ventricular septum and neural tube closure: general implications for tissue fusion processes. Development 2010: 137: 3707-3717.
49. Wang Y, Guo N, Nathans J. The role of Frizzled3 and Frizzled6 in neural tube closure and in the planar polarity of inner-ear sensory hair cells. J Neurosci 2006: 26: 2147-2156.
50. Juriloff DM, Harris MJ. A consideration of the evidence that genetic defects in planar cell polarity contribute to the etiology of human neural tube defects. Birth Defects Res A Clin Mol Teratol 2012: 94: 824-840.
51. Pai YJ, Leung KY, Savery D et al. Glycine decarboxylase deficiency causes neural tube defects and features of non-ketotic hyperglycinemia in mice. Nat Commun 2015: 6: 6388.
52. Bassuk AG, Muthuswamy LB, Boland R et al. Copy number variation analysis implicates the cell polarity gene glypican 5 as a human spina bifida candidate gene. Hum Mol Genet 2013: 22: 1097-1111.
53. Li X, Roszko I, Sepich DS et al. Gpr125 modulates Dishevelled distribution and planar cell polarity signaling. Development 2013: 140: 3028-3039.
54. Gustavsson P, Copp AJ, Greene ND. Grainyhead genes and mammalian neural tube closure. Birth Defects Res A Clin Mol Teratol 2008: 82: 728-735.
55. Ting SB, Wilanowski T, Auden A et al. Inositol- and folate-resistant neural tube defects in mice lacking the epithelial-specific factor Grhl-3. Nat Med 2003: 9: 1513-1519.
56. Davidson CM, Northrup H, King TM et al. Genes in glucose metabolism and association with spina bifida. Reprod Sci 2008: 15: 51-58.
57. Jacoby M, Cox JJ, Gayral S et al. INPP5E mutations cause primary cilium signaling defects, ciliary instability and ciliopathies in human and mouse. Nat Genet 2009: 41: 1027-1031.

58. Park TJ, Haigo SL, Wallingford JB. Ciliogenesis defects in embryos lacking inturned or fuzzy function are associated with failure of planar cell polarity and Hedgehog signaling. Nat Genet 2006: 38: 303-311.
59. Wilson MP, Hugge C, Bielinska M et al. Neural tube defects in mice with reduced levels of inositol 1,3,4-trisphosphate 5/6-kinase. Proc Natl Acad Sci U S A 2009: 106: 9831-9835.
60. Takahashi M, Kojima M, Nakajima K et al. Cardiac abnormalities cause early lethality of jumonji mutant mice. Biochem Biophys Res Commun 2004: 324: 1319-1323.
61. Copp AJ, Carvalho R, Wallace A et al. Regional differences in the expression of laminin isoforms during mouse neural tube development. Matrix Biol 2011: 30: 301-309.
62. Robinson A, Partridge D, Malhas A et al. Is LMNB1 a susceptibility gene for neural tube defects in humans? Birth Defects Res A Clin Mol Teratol 2013: 97: 398-402.
63. Wu M, Chen DF, Sasaoka T et al. Neural tube defects and abnormal brain development in F52-deficient mice. Proc Natl Acad Sci U S A 1996: 93: 2110-2115.
64. Rocha PP, Bleiss W, Schrewe H. Mosaic expression of Med12 in female mice leads to exencephaly, spina bifida, and craniorachischisis. Birth Defects Res A Clin Mol Teratol 2010: 88: 626-632.
65. Lemos MC, Harding B, Reed AA et al. Genetic background influences embryonic lethality and the occurrence of neural tube defects in Men1 null mice: relevance to genetic modifiers. J Endocrinol 2009: 203: 133-142.
66. Momb J, Lewandowski JP, Bryant JD et al. Deletion of Mthfd1l causes embryonic lethality and neural tube and craniofacial defects in mice. Proc Natl Acad Sci U S A 2013: 110: 549-554.
67. Jensen LE, Hoess K, Whitehead AS et al. The NAT1 C1095A polymorphism, maternal multivitamin use and smoking, and the risk of spina bifida. Birth Defects Res A Clin Mol Teratol 2005: 73: 512-516.
68. Deak KL, Boyles AL, Etchevers HC et al. SNPs in the neural cell adhesion molecule 1 gene (NCAM1) may be associated with human neural tube defects. Hum Genet 2005: 117: 133-142.
69. Pallerla SR, Pan Y, Zhang X et al. Heparan sulfate Ndst1 gene function variably regulates multiple signaling pathways during mouse development. Dev Dyn 2007: 236: 556-563.
70. Bose K, Nischt R, Page A et al. Loss of nidogen-1 and -2 results in syndactyly and changes in limb development. J Biol Chem 2006: 281: 39620-39629.
71. Safra N, Bassuk AG, Ferguson PJ et al. Genome-wide association mapping in dogs enables identification of the homeobox gene, NKX2-8, as a genetic component of neural tube defects in humans. PLoS Genet 2013: 9: e1003646.
72. Stottmann RW, Berrong M, Matta K et al. The BMP antagonist Noggin promotes cranial and spinal neurulation by distinct mechanisms. Dev Biol 2006: 295: 647-663.
73. Epstein DJ, Vogan KJ, Trasler DG et al. A mutation within intron 3 of the Pax-3 gene produces aberrantly spliced mRNA transcripts in the splotch (Sp) mouse mutant. Proc Natl Acad Sci U S A 1993: 90: 532-536.
74. Wang F, Wang J, Guo J et al. PCMT1 gene polymorphisms, maternal folate metabolism, and neural tube defects: a case-control study in a population with relatively low folate intake. Genes Nutr 2013: 8: 581-587.
75. Zhu H, Wicker NJ, Volcik K et al. Promoter haplotype combinations for the human PDGFRA gene are associated with risk of neural tube defects. Mol Genet Metab 2004: 81: 127-132.

76. Tao H, Suzuki M, Kiyonari H et al. Mouse prickle1, the homolog of a PCP gene, is essential for epiblast apical-basal polarity. Proc Natl Acad Sci U S A 2009: 106: 14426-14431.
77. Tao H, Inoue K, Kiyonari H et al. Nuclear localization of Prickle2 is required to establish cell polarity during early mouse embryogenesis. Dev Biol 2012: 364: 138-148.
78. Chu CW, Ossipova O, Ioannou A et al. Prickle3 synergizes with Wtip to regulate basal body organization and cilia growth. Sci Rep 2016: 6: 24104.
79. Cai C, Shi O. Genetic evidence in planar cell polarity signaling pathway in human neural tube defects. Front Med 2014: 8: 68-78.
80. Huang Y, Roelink H, McKnight GS. Protein kinase A deficiency causes axially localized neural tube defects in mice. J Biol Chem 2002: 277: 19889-19896.
81. Cogram P, Hynes A, Dunlevy LP et al. Specific isoforms of protein kinase C are essential for prevention of folate-resistant neural tube defects by inositol. Hum Mol Genet 2004: 13: 7-14.
82. Au KS, Ashley-Koch A, Northrup H. Epidemiologic and genetic aspects of spina bifida and other neural tube defects. Dev Disabil Res Rev 2010: 16: 6-15.
83. Ellis T, Smyth I, Riley E et al. Patched 1 conditional null allele in mice. Genesis 2003: 36: 158-161.
84. Paudyal A, Damrau C, Patterson VL et al. The novel mouse mutant, chuzhoi, has disruption of Ptk7 protein and exhibits defects in neural tube, heart and lung development and abnormal planar cell polarity in the ear. BMC Dev Biol 2010: 10: 87.
85. Wang M, De Marco P, Merello E et al. Role of the planar cell polarity gene Protein tyrosine kinase 7 in neural tube defects in humans. Birth Defects Res A Clin Mol Teratol 2015: 103: 1021-1027.
86. Camerer E, Barker A, Duong DN et al. Local protease signaling contributes to neural tube closure in the mouse embryo. Dev Cell 2010: 18: 25-38.
87. Martinez S, Scerbo P, Giordano M et al. The PTK7 and ROR2 Protein Receptors Interact in the Vertebrate WNT/Planar Cell Polarity (PCP) Pathway. J Biol Chem 2015: 290: 30562-30572.
88. Bohm J, Buck A, Borozdin W et al. Sall1, sall2, and sall4 are required for neural tube closure in mice. Am J Pathol 2008: 173: 1455-1463.
89. Murdoch JN, Henderson DJ, Doudney K et al. Disruption of scribble (Scrb1) causes severe neural tube defects in the circletail mouse. Hum Mol Genet 2003: 12: 87-98.
90. Escobedo N, Contreras O, Munoz R et al. Syndecan 4 interacts genetically with Vangl2 to regulate neural tube closure and planar cell polarity. Development 2013: 140: 3008-3017.
91. Merte J, Jensen D, Wright K et al. Sec24b selectively sorts Vangl2 to regulate planar cell polarity during neural tube closure. Nat Cell Biol 2010: 12: 41-46; sup pp 41-48.
92. Deng S, Hirschberg A, Worzfeld T et al. Plexin-B2, but not Plexin-B1, critically modulates neuronal migration and patterning of the developing nervous system in vivo. J Neurosci 2007: 27: 6333-6347.
93. Zhai G, Gu Q, He J et al. Sept6 is required for ciliogenesis in Kupffer's vesicle, the pronephros, and the neural tube during early embryonic development. Mol Cell Biol 2014: 34: 1310-1321.
94. Yang X, Cheyette BN. SEC14 and spectrin domains 1 (Sestd1) and Dapper antagonist of catenin 1 (Dact1) scaffold proteins cooperatively regulate the Van Gogh-like 2 (Vangl2) four-pass transmembrane protein and planar cell polarity (PCP) pathway during embryonic development in mice. J Biol Chem 2013: 288: 20111-20120.

95. Hu M, Sun XJ, Zhang YL et al. Histone H3 lysine 36 methyltransferase Hypb/Setd2 is required for embryonic vascular remodeling. Proc Natl Acad Sci U S A 2010: 107: 2956-2961.
96. Misra K, Matise MP. A critical role for sFRP proteins in maintaining caudal neural tube closure in mice via inhibition of BMP signaling. Dev Biol 2010: 337: 74-83.
97. Satoh W, Matsuyama M, Takemura H et al. Sfrp1, Sfrp2, and Sfrp5 regulate the Wnt/beta-catenin and the planar cell polarity pathways during early trunk formation in mouse. Genesis 2008: 46: 92-103.
98. Echelard Y, Epstein DJ, St-Jacques B et al. Sonic hedgehog, a member of a family of putative signaling molecules, is implicated in the regulation of CNS polarity. Cell 1993: 75: 1417-1430.
99. Beaudin AE, Abarinov EV, Noden DM et al. Shmt1 and de novo thymidylate biosynthesis underlie folate-responsive neural tube defects in mice. Am J Clin Nutr 2011: 93: 789-798.
100. Lee C, Le MP, Wallingford JB. The shroom family proteins play broad roles in the morphogenesis of thickened epithelial sheets. Dev Dyn 2009: 238: 1480-1491.
101. Hildebrand JD, Soriano P. Shroom, a PDZ domain-containing actin-binding protein, is required for neural tube morphogenesis in mice. Cell 1999: 99: 485-497.
102. Lemay P, Guyot MC, Tremblay E et al. Loss-of-function de novo mutations play an important role in severe human neural tube defects. J Med Genet 2015: 52: 493-497.
103. Berk M, Desai SY, Heyman HC et al. Mice lacking the ski proto-oncogene have defects in neurulation, craniofacial, patterning, and skeletal muscle development. Genes Dev 1997: 11: 2029-2039.
104. Li R, Thorens B, Loeken MR. Expression of the gene encoding the high-Km glucose transporter 2 by the early postimplantation mouse embryo is essential for neural tube defects associated with diabetic embryopathy. Diabetologia 2007: 50: 682-689.
105. Mao J, McKean DM, Warrier S et al. The iron exporter ferroportin 1 is essential for development of the mouse embryo, forebrain patterning and neural tube closure. Development 2010: 137: 3079-3088.
106. Narimatsu M, Bose R, Pye M et al. Regulation of planar cell polarity by Smurf ubiquitin ligases. Cell 2009: 137: 295-307.
107. Zheng B, Tang T, Tang N et al. Essential role of RGS-PX1/sorting nexin 13 in mouse development and regulation of endocytosis dynamics. Proc Natl Acad Sci U S A 2006: 103: 16776-16781.
108. Kase BA, Northrup H, Morrison AC et al. Association of copper-zinc superoxide dismutase (SOD1) and manganese superoxide dismutase (SOD2) genes with nonsyndromic myelomeningocele. Birth Defects Res A Clin Mol Teratol 2012: 94: 762-769.
109. Bauters M, Frints SG, Van Esch H et al. Evidence for increased SOX3 dosage as a risk factor for X-linked hypopituitarism and neural tube defects. Am J Med Genet A 2014: 164A: 1947-1952.
110. Szabo R, Hobson JP, Christoph K et al. Regulation of cell surface protease matriptase by HAI2 is essential for placental development, neural tube closure and embryonic survival in mice. Development 2009: 136: 2653-2663.
111. Szabo R, Uzzun Sales K, Kosa P et al. Reduced prostasin (CAP1/PRSS8) activity eliminates HAI-1 and HAI-2 deficiency-associated developmental defects by preventing matriptase activation. PLoS Genet 2012: 8: e1002937.
112. Godbole K, Gayathri P, Ghule S et al. Maternal one-carbon metabolism, MTHFR and TCN2 genotypes and neural tube defects in India. Birth Defects Res A Clin Mol Teratol 2011: 91: 848-856.

113. Schorle H, Meier P, Buchert M et al. Transcription factor AP-2 essential for cranial closure and craniofacial development. Nature 1996: 381: 235-238.
114. Bertolino E, Reimund B, Wildt-Perinic D et al. A novel homeobox protein which recognizes a TGT core and functionally interferes with a retinoid-responsive motif. J Biol Chem 1995: 270: 31178-31188.
115. Chih B, Liu P, Chinn Y et al. A ciliopathy complex at the transition zone protects the cilia as a privileged membrane domain. Nat Cell Biol 2011: 14: 61-72.
116. Abdelhamed ZA, Wheway G, Szymanska K et al. Variable expressivity of ciliopathy neurological phenotypes that encompass Meckel-Gruber syndrome and Joubert syndrome is caused by complex de-regulated ciliogenesis, Shh and Wnt signalling defects. Hum Mol Genet 2013: 22: 1358-1372.
117. Armstrong JF, Kaufman MH, Harrison DJ et al. High-frequency developmental abnormalities in p53-deficient mice. Curr Biol 1995: 5: 931-936.
118. Ikeda A, Ikeda S, Gridley T et al. Neural tube defects and neuroepithelial cell death in Tulp3 knockout mice. Hum Mol Genet 2001: 10: 1325-1334.
119. Chen ZF, Behringer RR. twist is required in head mesenchyme for cranial neural tube morphogenesis. Genes Dev 1995: 9: 686-699.
120. Nonn L, Williams RR, Erickson RP et al. The absence of mitochondrial thioredoxin 2 causes massive apoptosis, exencephaly, and early embryonic lethality in homozygous mice. Mol Cell Biol 2003: 23: 916-922.
121. Volcik KA, Shaw GM, Zhu H et al. Risk factors for neural tube defects: associations between uncoupling protein 2 polymorphisms and spina bifida. Birth Defects Res A Clin Mol Teratol 2003: 67: 158-161.
122. Kibar Z, Torban E, McDearmid JR et al. Mutations in VANGL1 associated with neural-tube defects. N Engl J Med 2007: 356: 1432-1437.
123. Torban E, Wang HJ, Groulx N et al. Independent mutations in mouse Vangl2 that cause neural tube defects in looptail mice impair interaction with members of the Dishevelled family. J Biol Chem 2004: 279: 52703-52713.
124. Andre P, Song H, Kim W et al. Wnt5a and Wnt11 regulate mammalian anterior-posterior axis elongation. Development 2015: 142: 1516-1527.
125. Qian D, Jones C, Rzadzinska A et al. Wnt5a functions in planar cell polarity regulation in mice. Dev Biol 2007: 306: 121-133.
126. Dabdoub A, Donohue MJ, Brennan A et al. Wnt signaling mediates reorientation of outer hair cell stereociliary bundles in the mammalian cochlea. Development 2003: 130: 2375-2384.
127. Rochard L, Monica SD, Ling IT et al. Roles of Wnt pathway genes wls, wnt9a, wnt5b, frzb and gpc4 in regulating convergent-extension during zebrafish palate morphogenesis. Development 2016: 143: 2541-2547.
128. Aruga J, Minowa O, Yaginuma H et al. Mouse Zic1 is involved in cerebellar development. J Neurosci 1998: 18: 284-293.
129. Nagai T, Aruga J, Minowa O et al. Zic2 regulates the kinetics of neurulation. Proc Natl Acad Sci U S A 2000: 97: 1618-1623.
130. Klootwijk R, Franke B, van der Zee CE et al. A deletion encompassing Zic3 in bent tail, a mouse model for X-linked neural tube defects. Hum Mol Genet 2000: 9: 1615-1622.
131. Carrel T, Purandare SM, Harrison W et al. The X-linked mouse mutation Bent tail is associated with a deletion of the Zic3 locus. Hum Mol Genet 2000: 9: 1937-1942.
132. Hao HX, Xie Y, Zhang Y et al. ZNRF3 promotes Wnt receptor turnover in an R-spondin-sensitive manner. Nature 2012: 485: 195-200.
